# A novel strategy for D-psicose and lipase co-production using a co-culture system of engineered *Bacillus subtilis* and *Escherichia coli* and bioprocess analysis using metabolomics

**DOI:** 10.1186/s40643-021-00429-8

**Published:** 2021-08-19

**Authors:** Jun Zhang, Wen Luo, Zhiyuan Wang, Xiaoyan Chen, Pengmei Lv, Jingliang Xu

**Affiliations:** 1 Guangdong Provincial Key Laboratory of New and Renewable Energy Research and Development, Guangzhou Institute of Energy Conversion, Chinese Academy of Sciences, CAS Key Laboratory of Renewable Energy, Guangzhou, 510640 China; 2 College of Food Science and Technology, Guangdong Provincial Key Laboratory of Aquatic Product Processing and Safety, Guangdong Province Engineering Laboratory for Marine Biological Products, Guangdong Provincial Engineering Technology Research Center of Seafood, Key Laboratory of Advanced Processing of Aquatic Product of Guangdong Higher Education Institution, Guangdong Ocean University, Zhanjiang, 524088 China; 3 School of Chemical Engineering, Zhengzhou University, Zhengzhou, 450001 China; 4 University of Chinese Academy of Sciences, Beijing, 100049 China

**Keywords:** D-Psicose, Lipase, *Escherichia coli*, *Bacillus subtilis*, Co-culture, Metabolomics

## Abstract

To develop an economically feasible fermentation process, this study designed a novel bioprocess based on the co-culture of engineered *Bacillus subtilis* and *Escherichia coli* for the co-production of extracellular D-psicose and intracellular lipase. After optimizing the co-culture bioprocess, 11.70 g/L of D-psicose along with 16.03 U/mg of lipase was obtained; the glucose and fructose were completely utilized. Hence, the conversion rate of D-psicose reached 69.54%. Compared with mono-culture, lipase activity increased by 58.24%, and D-psicose production increased by 7.08%. In addition, the co-culture bioprocess was explored through metabolomics analysis, which included 168 carboxylic acids and derivatives, 70 organooxygen compounds, 34 diazines, 32 pyridines and derivatives, 30 benzene and substituted derivatives, and other compounds. It also could be found that the relative abundance of differential metabolites in the co-culture system was significantly higher than that in the mono-culture system. Pathway analysis revealed that, tryptophan metabolism and *β*-alanine metabolism had the highest correlation and played an important role in the co-culture system; among them, tryptophan metabolism regulates protein synthesis and *β*-alanine metabolism, which is related to the formation of metabolic by-products. These results confirm that the co-cultivation of *B. subtilis* and *E. coli* can provide a novel idea for D-psicose and lipase biorefinery, and are beneficial for the discovery of valuable secondary metabolites such as turanose and morusin.

**Supplementary Information:**

The online version contains supplementary material available at 10.1186/s40643-021-00429-8.

## Highlights

Co-cultivation was firstly carried out for the co-production of D-psicose and lipase.

The carbon source glucose and fructose were fully utilized during co-culture.

The yield of D- psicose was 11.70 g/L, and the conversion rate reached 69.54%.

Self-induced expression of lipase was realized with enzyme activity of 16.03 U/mg.

Metabolomics analyses were used to explore the co-culture effects of mixed strains.

**Supplementary Information:**

The online version contains supplementary material available at 10.1186/s40643-021-00429-8.

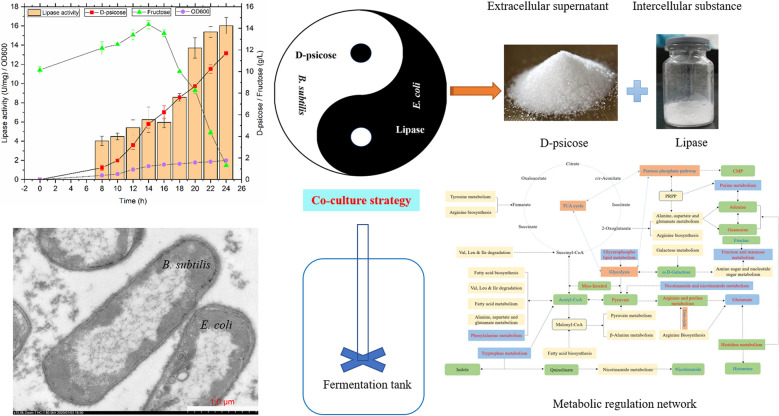

## Introduction

D-Psicose is a rare sugar and a new type of functional sweetener with excellent physiological effects such as anti-hyperglycemic and anti-hyperlipidemic effects (Chen et al. 2019; Zhang et al. 2016). Therefore, it is widely used in food, medicine, and other fields. Presently, enzymatic conversion is the most important method for the synthesis of D-psicose, and under the catalytic action of ketose 3-epimerases, D-fructose can be isomerized into D-psicose, which is the current hot spot of D-psicose synthesis (Jiang et al. 2020). However, the use of fermentation methods for the cultivation of engineered *Bacillus subtilis*, which harbors the D-tagatose 3-epimerase (DTEase) gene, has the potential to directly produce D-psicose as reported in a previous study (Zhang et al. 2020a). The conversion rate of the substrate can reach 56.26%, which is a great advantage in realizing the coupling of cell growth and D-psicose production, thereby reducing the biosynthesis costs.

Lipase is a hydrolase which acts on the ester bond with catalytic diversity, and it is also one of the most widely used biocatalysts in industrial production (Melani et al. 2020). *Geobacillus thermocatenulatus* lipase 2 (GTL2) is a typical thermophilic enzyme with high catalytic activity and good thermal stability and has several applications in pharmaceutical and organic synthesis, chiral compound resolution, and bioenergy; thus, it is a promising biocatalyst with great application potential (Godoy et al. 2019; Kajiwara et al. 2020). In addition, *Escherichia coli* has a short culture cycle with high target protein levels, which makes it a good host for expressing GTL2. Under the induction of expensive IPTG, GTL2 achieved soluble expression in *E. coli*, and the enzyme activity reached 39.50 U/mg in a previous study (Zhang et al. 2020b). However, to enhance the fermentation economy, it is desirable to induce GTL2 expression using *α*-lactose.

Presently, single-bacterial fermentation of lipase may cause excessive production costs, whereas mixed-bacterial fermentation of expensive D-psicose can reduce the fermentation costs. This innovation in traditional fermentation not only reduces the overall cost, but also improves the quality of fermentation. In addition, co-culture bioprocess can also effectively utilize glucose and fructose, which is of relevance for complex substrates, such as waste fruit and vegetable hydrolysate (Jiang et al. 2017), to achieve the goal of carbon neutrality, and the full consumption of the carbon source is conducive to the subsequent separation of D-psicose. Furthermore, in previous studies, the extracellular production of D-psicose in recombinant *B. subtilis* (Zhang et al. 2020a) and the soluble expression of GTL2 in engineered *E. coli* (Zhang et al. 2020b) have been achieved. Hence, it is better to explore the possibility of co-production of D-psicose and lipase using co-culture to develop a more economical fermentation process.

Based on this principle of symbiosis (Wein et al. 2019), the co-culture of microorganisms is used to ferment mixed microorganisms with complementary advantages (Bertrand et al. 2014; Rosero-Chasoy et al. 2021). Furthermore, it has a strong ability to tolerate harsh environments, such as infection, substrate, and product inhibition; hence, better fermentation results can be obtained. Currently, there are two main forms of co-culture systems (Wein et al. 2019): (a) the co-cultivation of unknown microbial flora isolated from nature and (b) the co-cultivation of artificially designed microbial flora. Compared with the former, the latter system is relatively fragile; therefore, there should be a careful selection of the combined strains. To ensure the efficient and stable growth of the co-culture system, the population ratio in a reasonable range and external conditions at certain stages, which are favored by priority populations, has been maintained through optimized medium components and strengthened process regulation (Klitgord and Segre 2010). *B. subtilis* and *E. coli* are Gram-positive and Gram-negative bacteria, respectively. They have a well-mapped genetic background and are commonly used as model organisms. Under optimized co-culture conditions, they can produce effective synergistic effects and increase the yield of target products (Faust and Raes 2012). Recently, indole (an aromatic heterocyclic organic compound) was produced by co-cultivation of *E. coli* and *B. subtilis* with significantly improved yield (Singh et al. 2020).

The construction of an artificial co-culture system is relatively easy and simple, but metabolic regulation is relatively complicated. Thus, it is necessary to use metabolomics analysis to explore the symbiotic effects of mixed strains. In general, metabolomics is defined as the study of the overall metabolite spectrum in a system (cell, tissue, or organism) under given conditions. It is a collection of small molecular metabolites with a molecular weight of less than 1000 in biological samples (Patti et al. 2012). A large amount of qualitative and quantitative metabolite data can be obtained and classified according to specific metabolic pathways to construct metabolic networks and infer their physiological functions to be able to more comprehensively analyze the dynamic metabolic reactions of organisms in response to environmental changes. Microbial metabolites, as well as the identification of metabolic variations in different environments, can easily be screened through effective analytical methods, such as liquid chromatography–mass spectrometry (LC–MS) based on the UHPLC–QE-MS nontargeted metabolomics approach (Cheng et al. 2020; Xia et al. 2020). However, there is limited information about the differential metabolites and key metabolic pathways in the co-culture process of *B. subtilis* and *E. coli* in the existing literature. Hence, the screening of major differential metabolites and metabolic pathways using metabolomics analysis can better manipulate the co-cultivation process of *B. subtilis* and *E. coli*, which is beneficial to the stable operation of the co-culture system.

In this study, a co-culture system of recombinant *B. subtilis* and *E. coli* was developed to explore the co-production of D-psicose and lipase. The feasibility of co-production by bacterial cultivation was verified through shake-flask cultures, and the mixed strains using a fermentation tank to increase D-psicose production and lipase activity were also carried out. With the help of metabolomics, a comparative analysis was performed to evaluate the metabolites of the co-culture or mono-culture. Combined with the KEGG database and network analysis, the metabolic pathways that played an important role in the bioprocess were also investigated to clarify the metabolic regulation of the artificial co-culture system.

## Materials and methods

### Strains and media

In this study, engineered *B. subtilis* (Zhang et al. 2020a) and *E. coli* (Zhang et al. 2020b) were used to produce D-psicose and GTL2, respectively, through a co-culture bioprocess. Luria–Bertani (LB) medium containing appropriate antibiotics (50 µg/mL kanamycin) was used as a seed medium, which consisted of tryptone (10 g/L), yeast extract (5 g/L), and NaCl (10 g/L) (Sezonov et al. 2007). The fermentation medium (Studier 2005) comprised yeast extract (15 g/L), glucose (10 g/L), fructose (10 g/L), *α*-lactose (2 g/L), NaCl (8 g/L), KH_2_PO_4_ (3.40 g/L), Na_2_HPO_4_ (3.55 g/L), (NH_4_)_2_SO_4_ (6.61 g/L), MgSO_4_•7H_2_O (0.71 g/L), MnCl_4_•4H_2_O (0.99 g/L), and kanamycin (50 µg/mL). All experimental materials were supplied by Sangon Biotech Co., Ltd. (Shanghai, China).

### Construction of co-culture system using engineered *B. subtilis* and *E. coli*

To explore the feasibility of co-fermentation of recombinant strains, 1 mL of seed cultures for recombinant * B. subtilis and E. coli* were transferred into a 500-mL shake-flask containing 200 mL of LB medium and incubated at 37 °C and 200 rpm for 24 h. A cell pellet was obtained through centrifugation (8000 × *g*, 5 min), which was washed with deionized water. Next, a suitable amount of bacterial solution was used to prepare a smear, and the distribution of these cells was analyzed using Gram staining. The resulting protein solution of bacterial co-culture through ultrasonic disruption of cell pellets was analyzed using sodium dodecyl sulfate polyacrylamide gel electrophoresis (SDS-PAGE). In addition, the mono-culture of the engineered *B. subtilis* and *E. coli* was prepared under the same conditions as the above co-culture, and the protein solution obtained was subjected to protein level analysis using SDS-PAGE.

### Lipase activity analysis

Lipase activity was determined using enzymatic hydrolysis of ρ-nitrophenol released from the substrate ρ-nitrophenyl palmitate in Tris–HCl buffer (50 mM, pH 8.0) at 60 °C. Under the detection conditions, the amount of enzyme required to release 1 µmol of ρ-nitrophenol through hydrolysis per unit time was defined as the unit enzyme activity (U/mg). Furthermore, optical density (OD_410_ and OD_562_) analysis was also carried out for the determination of ρ-nitrophenol and protein concentration according to a previous study (Zhang et al. 2020a).

### Optimization of co-culture conditions using shake-flask cultures

To optimize the co-production of D-psicose and lipase, the effects of fermentation factors such as time (0–32 h), temperature (28–40 °C), pH (6.0–9.0), D-fructose (5–30 g/L), *α*-lactose (0–10 g/L), and inoculation ratio (*B. subtilis*: *E. coli* = 4:1–1:4) were investigated. Glycerol stock strains (200 µL) were inoculated in a 500-mL Erlenmeyer flask containing 200 mL of LB medium and were cultured at 37 °C and 180 rpm for 16–18 h as seed cultures. Then, a certain inoculation ratio of engineered *B. subtilis* and *E. coli* (*B. subtilis*: *E. coli* = 2:1) was inoculated in a 250-mL Erlenmeyer flask containing 50 mL of fermentation medium, which was, then cultured at 37 °C and 200 rpm for 24 h. At the end of fermentation, the supernatant and cell pellets were collected. The supernatant was tested for the concentration of D-psicose, and the pellet was washed with deionized water and resuspended in Tris–HCl buffer (pH 8.0). Afterwards, the ultrasonication process was employed to prepare lipase solution.

### Co-production of D-psicose and lipase using fermentation tank

Seed cultures (3%) were transferred into two identical 1-L fermenters (Applikon Biotechnology, Netherlands) containing 500 mL of fermentation medium (pH 6.0) for batch cultivation, according to the 1:2 inoculation ratio of recombinant *B. subtilis* and *E. coli*, which was then kept at 37 °C and 200 rpm for 24 h. Meanwhile, sterile dry air was introduced at a rate of 3 L/min for 10 h during the fermentation process. For comparison with the co-fermentation of recombinant strains, the engineered *B. subtilis* and *E. coli* were cultured with an inoculum of 1% and 2%, respectively, under the same conditions.

### Transmission electron microscopy (TEM) and heteronuclear single quantum coherence (HSQC) analysis

TEM (FEI, Hitachi Ltd, Japan) analysis was carried out after taking appropriate recombinant cells fixed with 2.5% glutaraldehyde at the end of batch-fermentation, and this test was performed by Servicebio Co., Ltd (Wuhan, China); An appropriate amount of co-culture supernatant was processed with a freeze dryer, fully dissolved in deuterated water, and loaded into a nuclear magnet tube. Finally, HSQC was performed using nuclear magnetic resonance (AVANCE III, Bruker, Germany).

### Samples and metabolites extraction

The samples used for metabolomics analysis were obtained from the mono-culture of *B. subtilis* and *E. coli* and their co-culture (Additional file 1: Table S1). Each group contained four samples in duplicate, which were used for intracellular and extracellular metabolomics analyses. For extracellular metabolite collection, the culture medium was first mixed, and then a certain amount of medium was immediately centrifuged at 4 °C (1000 × *g*, 10 min). Afterwards, 500 µL of the supernatant was transferred into a new centrifuge tube, placed in liquid nitrogen to quench for 30 s, and then stored at − 80 °C. For intracellular metabolite collection, the OD_600_ of the bacteria was first measured to calculate the volume of culture medium required for 1 × 10^7^ cells and then centrifuged at 4 °C (1000 × *g*, 10 min). Next, the bacterial solution was separated; the supernatant and strain were removed, immersed in liquid nitrogen to quench for 30 s, thawed on ice, and then washed with 1 × PBS buffer (pre-cooled at 4 °C or 20 °C). PBS buffer was then removed by centrifugation and finally stored at  − 80 °C. The extraction methods for intracellular and extracellular metabolites were the same. Briefly, samples (100 µL) were extracted with cold acetonitrile and methanol (400 µL, 1:1), sonicated for 10 min in an ice bath, followed by incubation for 1 h at  −  40 °C and centrifugation at 4 °C (8000 × *g*, 15 min). Therefore, the supernatant obtained was used for LC–MS analysis. Because the analysis principle and step of the positive ion mode are the same as the negative ion mode with only slight differences in numerical values, in this study, the positive ion mode was used as an example to illustrate the metabolome data.

### Metabolomics data and statistical analysis

The raw data were converted to the mzXML format using ProteoWizard and processed with an in-house program, which was developed using R and based on XCMS, for peak detection, extraction, alignment, and integration (Dunn et al. 2011). Then an in-house MS2 database created by Shanghai Biotree Biotech Co., Ltd. (Shanghai, China) was used for metabolite annotation, and the cut-off for annotation was set at 0.3. The final dataset containing peak number, sample name, and normalized peak area was imported into SIMCA15.0.2 (Sartorius Stedim Data Analytics AB, Umea, Sweden) for principal component analysis (PCA) (Jolliffe and Cadima 2016) and orthogonal projections to latent structures-discriminate analysis (OPLS-DA) (Wiklund et al. 2008). The Student’s *t*-test (*p* < 0.05) was used to evaluate the variables (Saccenti et al. 2014). In addition, commercial databases, including KEGG (http://www.genome.jp/kegg/) and MetaboAnalyst (http://www.metaboanalyst.ca/) were used for the comprehensive analysis of metabolic networks (Kanehisa and Goto 2000; Xia et al. 2015). Furthermore, regulatory network analyses of differential metabolites were also performed (Picart-Armada et al. 2018).

### Analytic methods

The optical densities of OD_410_, OD_562_, and OD_600_ were analyzed using an Eon Microplate Reader (Gene Company Limited, Chai Wan, Hong Kong). Glucose, D-fructose, and D-psicose were quantitatively analyzed using high performance liquid chromatography (HPLC) equipped with a Waters RID-2414 detector and a Sugar-Pak I column (Waters, Milford, MA, US). The column was eluted with water at a flow rate of 0.4 mL/min at 90 °C. The concentrations of formic, acetic, and glycolic acids were detected using HPLC equipped with RID-2414 and PDA-2998 detectors, and a sugar park SH-1011 column (Waters, Milford, MA, US). The column was eluted with 5 mM sulfuric acid at a flow rate of 0.5 mL/min at 50 °C. Metabolomics analysis was performed by Shanghai Biotree biotech Co., Ltd. (Shanghai, China). Specifically, UPLC-Q-TOF–MS/Ms (Vanquish, Thermo Fisher Scientific) with a UPLC BEH Amide column (2.1 × 100 mm, 1.7 µm) was set at 30 °C and coupled to Q Exactive HFX mass spectrometer (Orbitrap MS, Thermo), which was used to assess the metabolites. The mobile phase consisted of 25 mmol/L ammonium acetate and 25 mmol/L ammonium hydroxide in water (pH = 9.75) (A) and acetonitrile (B). The analysis was performed with elution gradient as follows: 0–0.5 min, 95% B; 0.5–7.0 min, 95–65% B; 7.0–8.0 min, 65–40% B; 8.0–9.0 min, 40% B; 9.0–9.1 min, 40–95% B; and 9.1–12.0 min, 95% B. The auto-sampler temperature was 4 °C, and the injection volume was 2 µL.

## Results and discussion

### Optimization of the co-culture system of engineered *B. subtilis* and *E. coli*

**Fig. 1:**
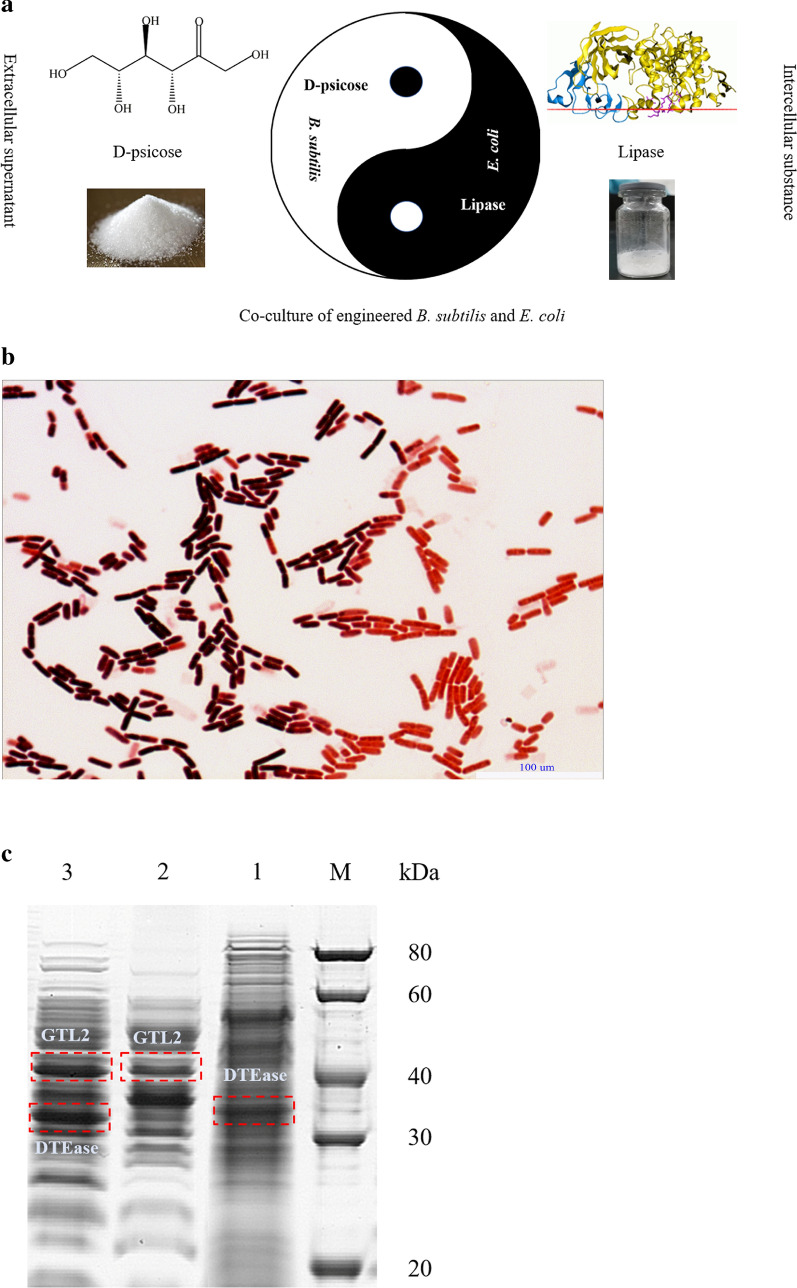
**a** Schematic diagram of D-psicose and lipase co-production using co-culture strategy; **b** Gram staining methods to identify mixed strains; **c** SDS-PAGE analysis of protein level in co-culture system. (Line M, standard protein band; Line 1, the protein level band of recombinant *B. subtilis*; 2, the protein level band of recombinant *E. coli*; 3, the protein level band of recombinant *B. subtilis* and *E. coli*.)

Intracellular lipase and extracellular D-psicose production have been achieved using recombinant strains in previous studies (Zhang et al. 2020a, b). Therefore, in this study, to develop a more economical fermentation process, co-cultivation of engineered *B. subtilis* and *E. coli* for the co-production of D-psicose and lipase was used (Fig. 1a). The recombinant strains in the co-culture system were analyzed using Gram staining analysis as shown in Fig. 1b. In the co-culture process, *B. subtilis* and *E. coli* were evenly distributed, and the purple and red staining results were more prominent compared with that observed in mono-culture process showing obvious characteristics of Gram-positive and negative bacteria. This result suggests that the artificially constructed co-culture system of engineered *B. subtilis* and *E. coli* could exist stably and grow normally. In addition, it can be seen from Fig. 1c that the protein level bands of recombinant DTEase and GTL2 became wider and darker after co-cultivation of *B. subtilis* and *E. coli*, which showed that the expression of the target protein increased. It was also confirmed that the mixed bacteria had a certain synergistic effect on cell growth via the co-culture bioprocess, which could effectively increase the cell concentration, thereby increasing the protein content.

**Fig. 2:**
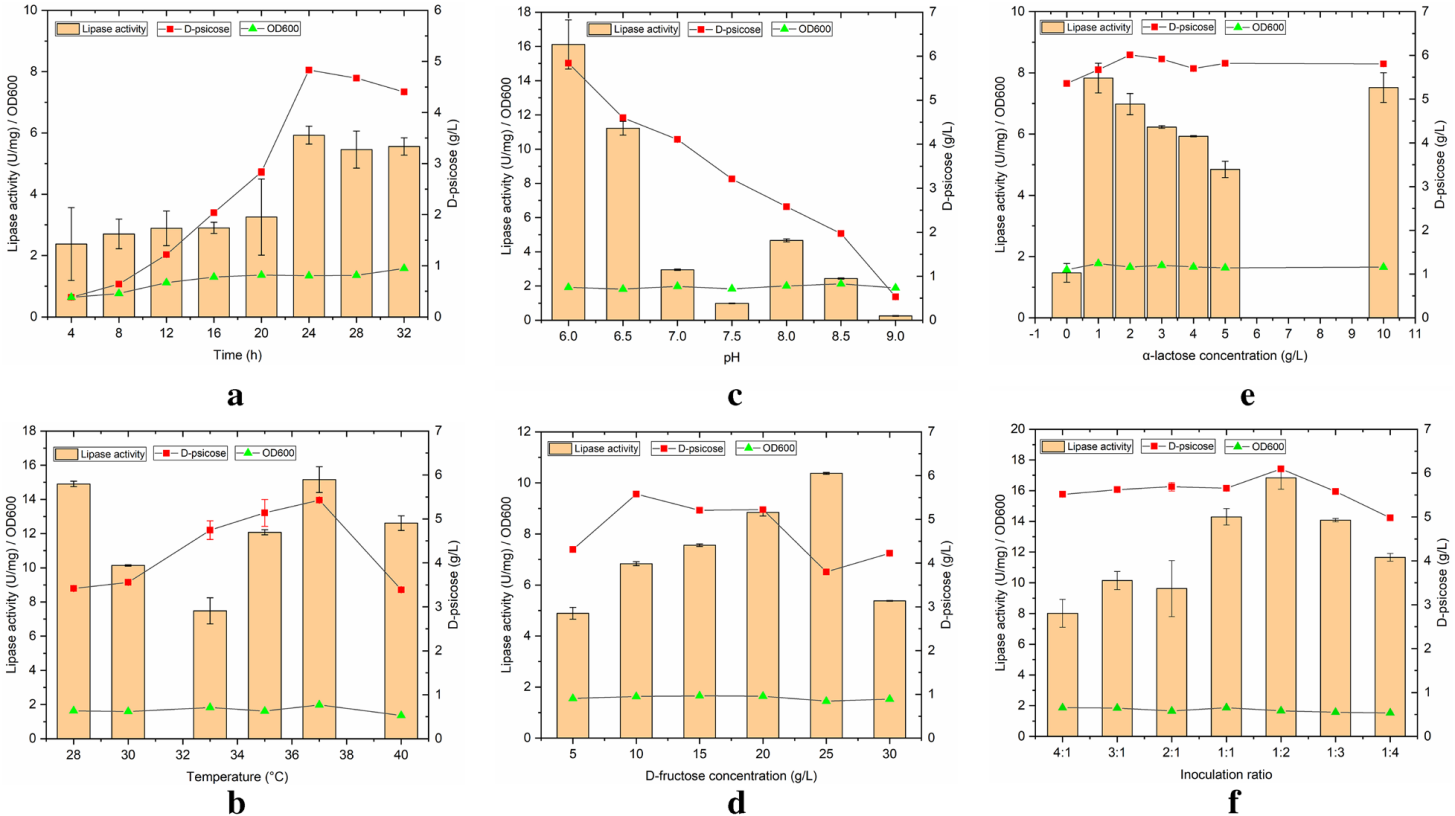
Effects of co-culture conditions on the co-production of D-psicose and lipase. **a** fermentation time (2 g/L *α*-lactose, 10 g/L D-fructose, initial pH 6.0, 37 °C, 32 h, and inoculation ratio of recombinant *B. subtilis* and *E. coli* = 1:2); **b** culture temperature (2 g/L *α*-lactose, 10 g/L D-fructose, initial pH 6.0, 24 h, and inoculation ratio of recombinant *B. subtilis* and *E. coli* = 1:2); **c** initial pH (2 g/L *α*-lactose, 10 g/L D-fructose, 37 °C, 24 h, and inoculation ratio of recombinant *B. subtilis* and *E. coli* = 1:2); **d** concentration of D-fructose (2 g/L *α*-lactose, initial pH 6.0, 37 °C, 24 h, and inoculation ratio of recombinant *B. subtilis* and *E. coli* = 1:2); **e** concentration of *α*-lactose (10 g/L D-fructose, initial pH 6.0, 37 °C, 24 h, and inoculation ratio of recombinant *B. subtilis* and *E. coli* = 1:2); **f** inoculation ratio of recombinant *B. subtilis* and *E. coli* (2 g/L *α*-lactose, 10 g/L D-fructose, initial pH 6.0, 37 °C, and 24 h)

As depicted in Fig. 2a, within 0–24 h, lipase activity, D-psicose, and cell concentration, increased with the extension of fermentation time, and when the time exceeded 24 h, cell growth of recombinant strains declined due to the limited growing space and nutrients. Thus, the best fermentation time was selected as 24 h, and 4.83 g/L of D-psicose together with 5.93 U/mg of lipase activity were obtained.

As shown in Fig. 2b, temperature had a significant effect on the co-culture process. This might be because the effects of temperature on co-culture not only affected the growth of recombinant cells, but also restricted the isomerization between fructose and D-psicose. In addition, temperature also affected the expression of recombinant lipase. Thus, the optimal fermentation temperature was 37 °C.

The effects of the initial pH of the medium on the co-culture bioprocess are depicted in Fig. 2c, which resemble the effects of temperature. The relatively low pH was beneficial to the increase in lipase activity and D-psicose production, with an initial pH of 6.0. This is because the initial pH is not only closely related to enzyme activity, but also affects the absorption of external substances by cells (Fang and Liu 2002). Therefore, it was inferred that an acidic environment was conducive to the synergistic promotion of the mixed strains. Thus, D-psicose production and lipase activity were the highest at an initial pH of 6.0.

The effects of fructose concentration are demonstrated in Fig. 2d. Fructose was used as a carbon source and as a reaction substrate during the co-fermentation process. However, the lipase activity and D-psicose showed varying trends at different fructose concentrations. In the range of 5–10 g/L, D-psicose production was enhanced with an increase in fructose concentration, and then with the increase in fructose concentration, D-psicose production showed a downward trend. There was a threshold for the isomerization reaction, which is catalyzed by epimerase; however, too much fructose might be required to promote the isomerization reaction causing an inhibitory effect to some extent. In contrast, in the range of 5–25 g/L, lipase activity was improved with an increase in fructose concentration. This may be due to *E. coli* preferring fructose as a carbon source. The highest lipase activity was 10.37 U/mg. Meanwhile, D-psicose production (3.80 g/L) was found to be lower. This decrease strongly proved that, in the presence of an abundant quantity of fructose, *E. coli* attained a more competitive advantage than *B. subtilis*. Subsequently, the reduction in enzymatic activity occurred due to the decline in *E. coli*. Considering the fermentation costs in the later stage, we used a 10 g/L concentration of fructose, 5.58 g/L of D-psicose, and 6.83 U/mg of lipase activity.

*α*-Lactose was used to achieve the self-induced expression of lipase to avoid the expensive inducer IPTG (Crowley and Rafferty 2019). The effect of *α*-lactose on the co-culture is shown in Fig. 2e. Varying the concentration of *α*-lactose had little effect on D-psicose production; when 2 g/L of *α*-lactose was used, 6.01 g/L of D-psicose and 6.98 U/mg of lipase activity was obtained. The concentration of *α*-lactose between 1 and 5 g/L has demonstrated a greater influence on lipase activity, as the increase in lactose concentration is related to a downward trend in the lipase activity. The *α*-lactose entered the cells directly under the action of the *α*-lactose permeating enzyme, and was converted into allolactose by galactosidase to act as an inducer. However, an increased concentration of *α*-lactose inhibits the regulation process of induction, which leads to a reduction in lipase activity.

According to a previous study (Zhang et al. 2020a), a 3% inoculum was selected during the co-cultivation of mixed strains. *B. subtilis* is a typical aerobe; therefore, it has a more obvious survival advantage than *E. coli* in the co-culture system. Thus, the inoculation ratio does not have a strong crucial effect on D-psicose production as compared to lipase activity. When the inoculation ratio of *B. subtilis* and *E. coli* was between 4:1 and 1:2, the inoculation amount of *B. subtilis* gradually decreased and that of *E. coli* steadily increased. D-Psicose production and especially lipase activity showed an increased variation (Fig. 2f). However, as the amount of *E. coli* inoculation continued to increase, D-psicose production and lipase activity both showed a downward trend. Therefore, the optimal inoculation ratio of mixed strains was chosen as 1:2 (*B. subtilis*: *E. coli*). Among them, D-psicose production was significantly higher than in the mono-culture of recombinant *B. subtilis* in a previous study (4.56 g/L) (Zhang et al. 2020a). These results confirmed that the synergistic effects of the co-culture had a reasonable range for the inoculation ratio. In the co-cultivation system, the competitive effects between *B. subtilis* and *E. coli* were the first, to balance this competitive effect and achieve synergistic effects, it is necessary to appropriately increase the amount of inoculated *E. coli* to enhance its competitiveness (Klitgord and Segre 2010). It was concluded that, when a competitive balance was reached in the co-cultivation system, a certain synergistic promotion effect would be manifested, thereby effectively increasing the production of D-psicose and lipase (Scafa et al. 2020).

### Co-production of D-psicose and lipase using fermentation tank

A fermentation tank was used for scale-up co-fermentation according to the parameters of the early-stage shake-flask co-cultures. To compare the advantages of co-fermentation of engineered *B. subtilis* and *E. coli*, fermentation of single bacteria was also carried out.

**Fig. 3:**
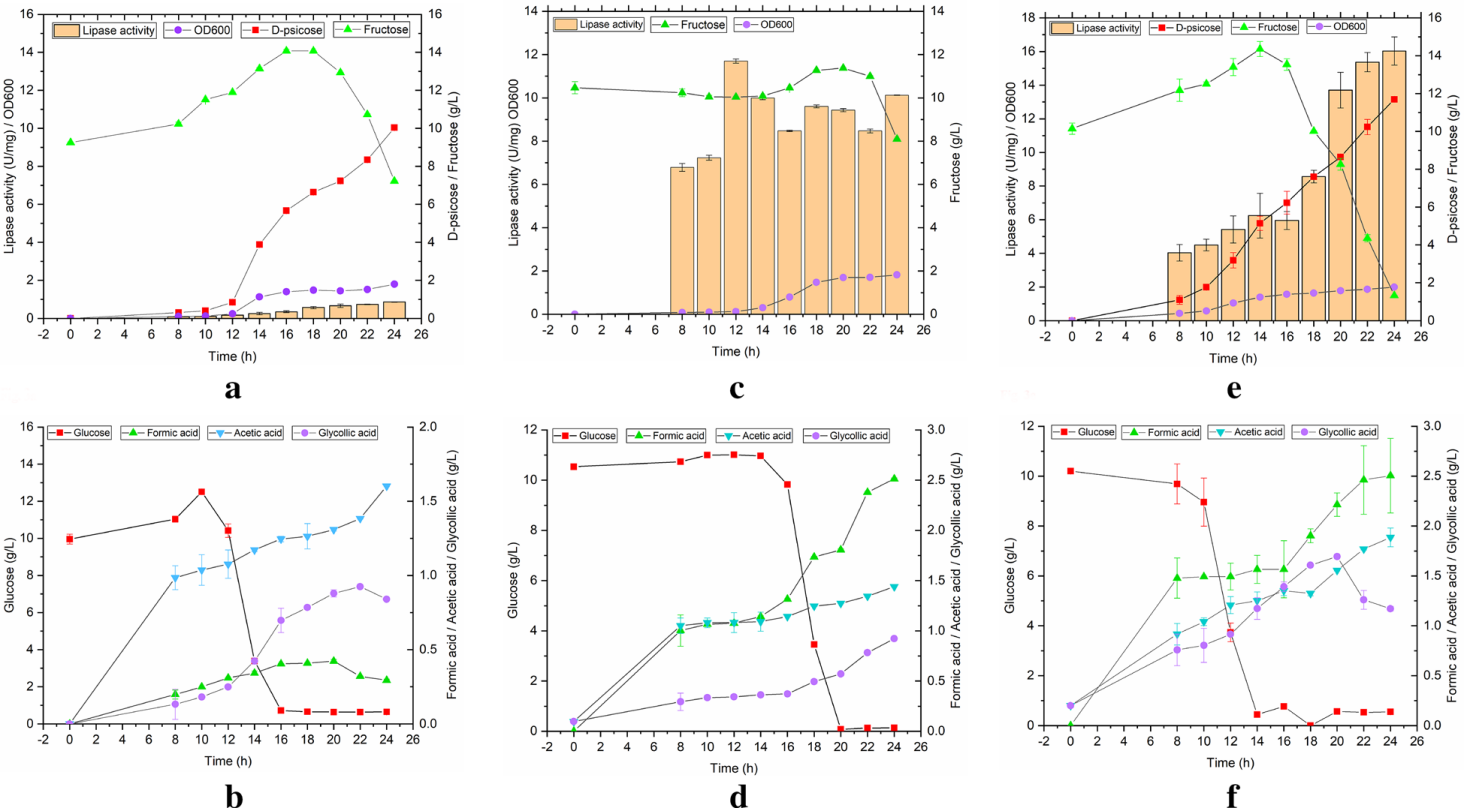
Batch fermentation of engineered *B. subtilis* (**a**, **b**), *E. coli* (**c**, **d**), *B. subtilis* and *E. coli* (**e**, **f**) using fermentation tank

The fermentation characteristics of recombinant *B. subtilis* are shown in Fig. 3a, b, and the OD_600_ was 1.795. During the fermentation process, glucose was nearly completely used up at 16 h, and fructose dropped sharply at 18 h, however, 7.22 g/L of fructose was retained until the end of fermentation; meanwhile, 10.04 g/L of D-psicose and 0.86 U/mg of lipase activity was obtained. Furthermore, the gradual increase in lipase activity was associated with the growth of *B. subtilis.* Potentially, *B. subtilis* could harbor the lipase gene (Sanchez et al. 2002), which could be translated and expressed under normal conditions with relatively low enzyme activity (0.86 U/mg). The by-product acetic acid increased with the extension of fermentation time, and the concentration was much higher than that of glycolic and formic acids; glycolic and formic acids started to decrease at 22 and 20 h, respectively. The final concentrations of cumulative metabolic by-products of acetic, glycolic, and formic acids were 1.60, 0.80, and 0.29 g/L, respectively.

The fermentation process of recombinant *E. coli* is shown in Fig. 3c, d, and the OD_600_ was 1.82. The variation trend in the carbon sources was as follows: glucose dropped rapidly after 14 h until it was nearly completely used up (0.13 g/L); fructose showed a gradual decrease at first and then increased, and finally decreased rapidly after 22 h. The strain expressed lipase through self-induction using *α*-lactose, and lipase activity showed an increased irregular trend during the fermentation process. The maximum lipase activity of 11.70 U/mg was achieved at 12 h, and then decreased or increased until the final enzyme activity was 10.13 U/mg. The by-products such as acetic, glycolic, and formic acids gradually increased with the progress of fermentation, and the final cumulative concentrations were 1.39, 0.85, and 2.51 g/L, respectively. However, none of them showed a decreasing trend, which was different from the accumulation of metabolic by-products of *B. subtilis*.

From Fig. 3e, f, the co-fermentation of *B. subtilis* and *E. coli* significantly improved D-psicose production and lipase activity, and the cell concentration was also enhanced with an OD_600_ of 1.997. In the co-culture system, the production of D-psicose increased steadily towards the maximum concentration of 11.70 g/L, and the lipase activity showed an increasing trend to a maximum of 16.03 U/mg. The content of glucose decreased sharply from the 12 h until the consumption was completed (0.55 g/L); the content of fructose showed a slowly increasing trend within 0–14 h, and dropped rapidly after 14 h until it was almost consumed (1.08 g/L). The by-products acetic and formic acids showed an increasing trend, and finally accumulated at 2.11 and 2.51 g/L. The content of glycolic acid showed a decreasing trend after 20 h, and the final accumulated amount was 1.22 g/L. Furthermore, through the co-fermentation of engineered *B. subtilis* and *E. coli*, the effective utilization of glucose and fructose was realized, and glucose and fructose were consumed during co-fermentation. According to the produced D-psicose and consumed glucose and fructose, the conversion rate of D-psicose could reach 69.54% (the ratio of D-psicose concentration to total consumed sugar concentration), and the conversion rate in this study was much higher than the previously reported enzymatic conversion rate (29.64%) (Zhang et al. 2018). Compared with single-bacterial fermentation, lipase activity was increased by 58.24%, and D-psicose production was enhanced by 7.08%, suggesting that mixed-bacterial fermentation could greatly promote the increase in yield. Furthermore, these results indicate that the co-culture system of recombinant *B. subtilis* and *E. coli* is more beneficial for the growth of *E. coli*.

**Fig. 4:**
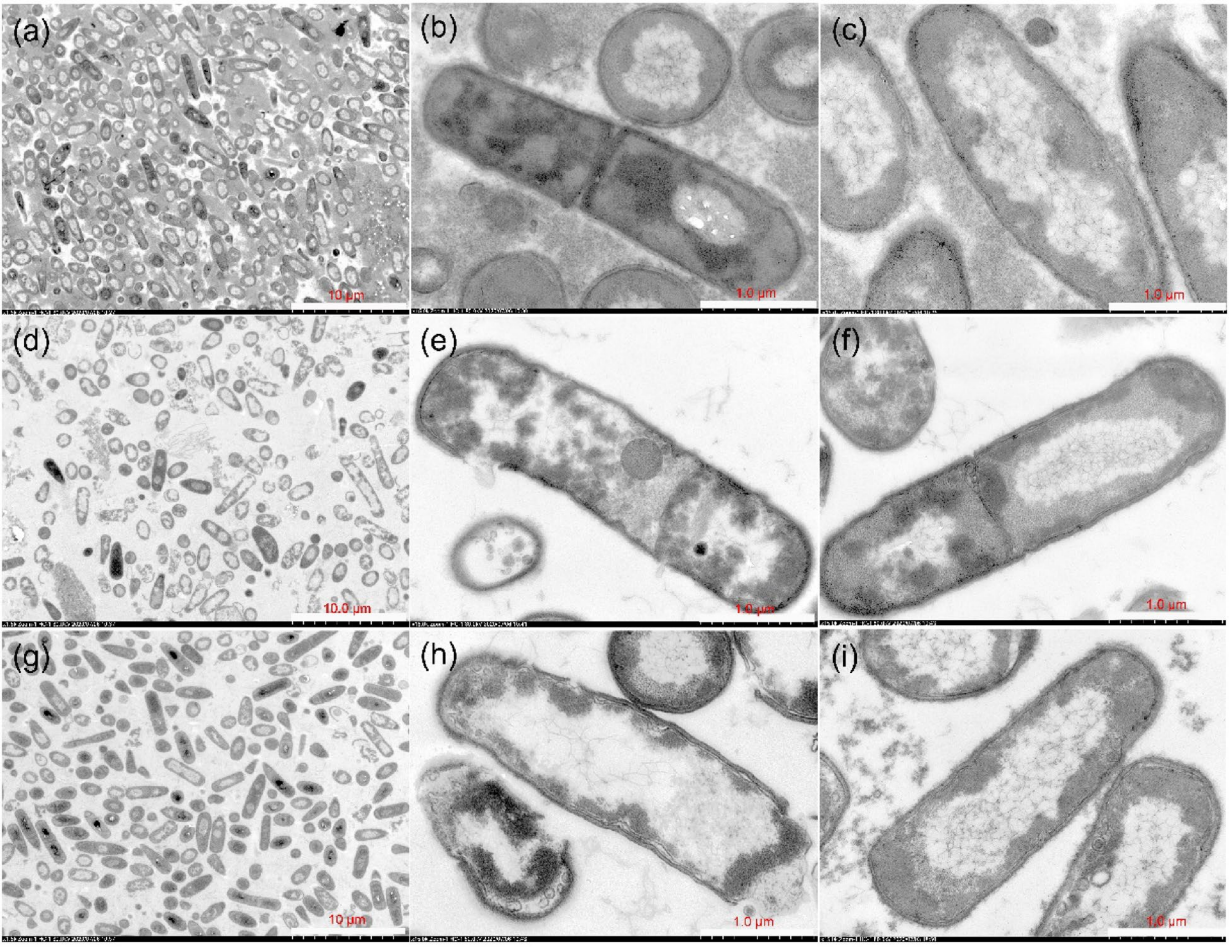
TEM analysis of recombinant *B. subtilis* (**a**–**c**), recombinant *E. coli* (**d**–**f**), and co-culture of recombinant *B. subtilis* and *E. coli* (**g**–**i**)

**Fig. 5:**
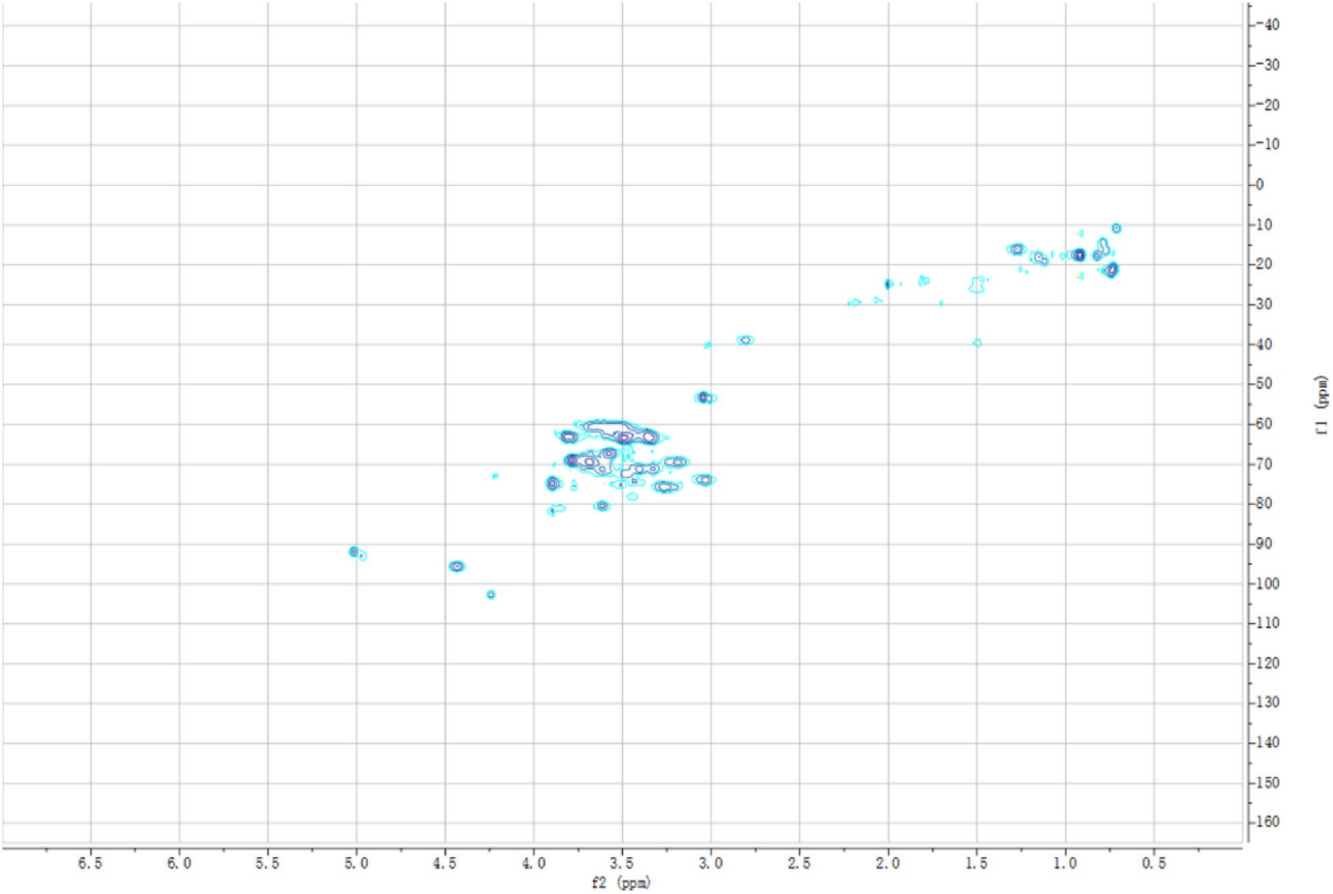
HSQC analysis of the fermentation supernatant of mixed strains by co-cultivation

The co-cultivation and mono-culture of recombinant strains are illustrated using TEM as shown in Fig. 4. *B. subtilis* and *E. coli* were evenly distributed in space and the strain density was more reasonable; however, *B. subtilis* grew densely, whereas *E. coli* was found sparsely within the mono-culture system. These results indicate that co-cultivation had a certain effect on the cell structure, which might be because some substances secreted during the bioprocess would directly act on the cell, such as the antimicrobial peptide (van Tilburg et al. 2020), secreted by *B. subtilis* which might damage the cell wall and improve its permeability. In addition, the HSQC analysis of co-fermentation supernatant is also presented in Fig. 5, which showed that the chemical shift of the glycoside proton was between 4.2 and 5.0 ppm, and the remaining protons were mainly concentrated between 4.0 and 3.0 ppm and approximately 2.0 and 1.0 ppm; the anomeric carbon was between 50 and 80 ppm. By comparing the HSQC characteristic spectra of standard D-psicose, D-fructose, and D-glucose (Additional file 1: Fig. S1), it is evident that most of the supernatant products were the target products of D-psicose. The contents of glucose and fructose were relatively low, which proved that the co-cultivation system of *E. coli* and *B. subtilis* had realized the effectively utilized carbon sources, which provided a novel idea for the utilization of carbon sources, especially the comprehensive utilization of fruit and vegetable wastes. Furthermore, it was helpful to realize the carbon cycle to prepare high value-added biochemicals while protecting the ecological environment.

Therefore, these beneficial results confirmed that the strategy of co-culture bioprocess with recombinant *B. subtilis* and *E. coli* was effective and constructive, and the synergistic effect shown in the co-culture system could provide a novel approach for biorefinery.

### Differential analyses of intracellular and extracellular metabolites

#### Differential metabolites analysis

In this study, 13,095 peaks were detected and left after relative standard deviation denoising. After the data were transformed and processed, PCA and OPLS-DA analyses were performed, and the model parameters are shown in Additional file 1: Table S2. Further, the OPLS-DA permutation test showed that the established models were more in line with the situation of the samples, which could well explain the gap between the two sets, and the original model had good robustness without overfitting (Additional file 1: Fig. S2–S6). The card value standard used in this study was that the *p*-value of the Student’s *t*-test was less than 0.05 and the variable importance in the projection (VIP) of the first principal component of the OPLS-DA was more than 1 to screen differential metabolites, and 684 metabolites were left after multivariate statistical analysis. The screened results of main differential metabolites are shown in Table 1 in the first 20 rows. Furthermore, the screened differential metabolites included 168 carboxylic acids and derivatives, 70 organooxygen compounds, 34 diazines, 32 pyridines and derivatives, 30 benzene and substituted derivatives, 27 fatty acids, 25 azoles, 22 pyrazines, 18 prenol lipids, 16 carbonyl compounds, 7 quinolines and derivatives, 3 flavonoids, and other compounds.

**Table 1:** The screened top 20 differential metabolites of engineered *B. subtilis* and *E. coli* during co-fermentation

Differential metabolites^a^	1 vs5	3 vs5	2 vs6	4 vs6
1	Choline	L-Proline	L-Proline	L-Proline
2	Adenine	Morusin	Choline	Choline
3	Turanose	Choline	Adenine	7,8-Dihydro-3b,6a-dihydroxy-alpha-ionol 9-glucoside
4	L-Histidine	Cellobiose	L-Histidine	1-Pyrroline
5	Isopropylpyrazine	7,8-Dihydro-3b,6a-dihydroxy-alpha-ionol 9-glucoside	2,5-Dihydro-2,4,5-trimethyloxazole	T2 Triol
6	Niacinamide	1-Pyrroline	Niacinamide	Protoanemonin
7	Gerberinol	T2 Triol	Gerberinol	Beta-Carboline
8	1H-Indole-3-carboxaldehyde	Protoanemonin	2,6-Diaminohexanoic acid	3-Methyladenine
9	Cytidine monophosphate	5H-Cyclopentapyrazine	1H-Indole-3-carboxaldehyde	Trimethylpyrazine
10	gamma-Aminobutyric acid	Beta-Carboline	L-Gulose	Dictyoquinazol C
11	N-Acetylcadaverine	Trehalose	Cytidine monophosphate	2-Ethyl-6-methylpyrazine
12	Histamine	4-Aminophenol	gamma-Aminobutyric acid	2-Methyl-3-propylpyrazine
13	N-Methylcalystegine B2	Turanose	N-Acetylcadaverine	2,5-Dihydro-2,4,5-trimethyloxazole
14	L-Histidinol	3-Methyladenine	Histamine	Adenosine
15	2'-O-Methyladenosine	(1S,2S,4R)-1,8-Epoxy-p-menthan-2-ol glucoside	N-Methylcalystegine B2	2,5-Dihydro-2,4-dimethyloxazole
16	Indoleacetaldehyde	Trimethylpyrazine	( +)-2,3-Dihydro-3-methyl-1H-pyrrole	Niacinamide
17	Levan	Dictyoquinazol C	Methenamine	Gerberinol
18	1-Pyrroline-5-carboxylic acid	2-Ethyl-6-methylpyrazine	gamma-Glutamyltyrosine	beta-Alanine
19	Alanyl-proline	2-Methyl-3-propylpyrazine	2'-O-Methyladenosine	4-Hydroxy-2-butenoic acid gamma-lactone
20	(Â ±)-2-Methylthiazolidine	2,5-Dihydro-2,4,5-trimethyloxazole	L-Pipecolic acid	2-[(5-Methylsulfinyl)-4-penten-2-ynylidene]-1,6-dioxaspiro[4.4]non-3-ene

^a^The card value standard for screening differential metabolites is *p* < 0.05 and VIP > 1.0

The quantitative value of the differential metabolites was calculated using Euclidean distance matrix clustering differential metabolites through a complete interlocking method and displaying them on a heatmap (Rangel-Huerta et al. 2019). Additional file 1: Fig. S7 visually reflects the main extracellular and intracellular differential metabolites of *B. subtilis* and *E. coli* during the fermentation process by hierarchical cluster analysis. The red color indicates high expression of the metabolites, and the blue color indicates low expression of the metabolites. Therefore, differential metabolites with the same characteristics can be clearly observed in the figure. Additional file 1: Fig. S7a and c describe the extracellular metabolites, and Additional file 1: Fig. S7b and d represent the intracellular metabolites. The color blocks at different positions represent the relative expression levels of the metabolites at the corresponding positions. Thus, the relative content of most metabolites in the co-culture system was significantly higher than that in the mono-culture system. This suggests that the biochemical metabolism of recombinant strains during the co-fermentation process is more complicated.

The corresponding ratio for the quantitative value of differential metabolites was calculated, taking the logarithmic conversion with base 2, and displaying the variation of content using a radar chart (Lee et al. 2016), and the radar chart could clarify the corresponding trend of metabolite content (Additional file 1: Fig. S8). Compared with the mono-culture group, in the co-culture group, the extracellular metabolites such as choline, adenine, turanose, L-histidine isopropylpyrazine, niacinamide, gerberinol, 1H-indole-3-carboxaldehyde, cytidine, monophosphate, gamma-aminobutyric acid, L-proline, morusin, cellobiose, 7,8-dihydro-3b,6a-dihydroxy-alpha-ionol 9-glucoside, 1-pyrroline, T2 triol, protoanemonin, and 5H-cyclopentapyrazine were found in relatively abundant quantities. The intracellular metabolite consisted of L-proline, choline, adenine, L-histidine, 2,5-dihydro-2,4,5-trimethyloxazole, niacinamide, gerberinol, 2,6-diaminohexanoic acid, 1H-indole-3-carboxaldehyde, 7,8-dihydro-3b,6a-dihydroxy-alpha-ionol 9-glucoside, 1-pyrroline, T2 triol, protoanemonin, beta-carboline, 3-methyladenine, trimethylpyrazine, and dictyoquinazol C, which were present in relatively high quantities. These results suggest that the extracellular metabolites of turanose, and morusin found during the co-fermentation were of constructive significance. Among them, turanose has physical and chemical properties similar to those of sucrose but is low in calories, which is expected to become a novel functional sweeter instead of sucrose (Seo et al. 2020). This is the first time that turanose production has been found in the co-culture system of *B. subtilis* and *E. coli*. It is also the first time that the flavonoid morusin is found in the co-cultivation process of recombinant strains, and it has potential medical application value (Choi et al. 2020).

Additional file 1: Fig. S9 shows the correlation analysis of differential metabolites, which is used to measure the closeness of the correlation between different metabolites and is quantitatively described by the correlation coefficient. The horizontal and vertical coordinates in the figure represent the different metabolites of the comparison, and the color blocks at different positions represent the correlation coefficient between the metabolites at the corresponding position. Red indicates a positive correlation, blue indicates negative correlation, a darker color indicates, a stronger correlation, and non-significant correlations are indicated by crosses. Through correlation analysis, the degree of correlation of differential metabolites in the co-culture process can be vividly displayed. The intracellular metabolites were found to be more closely related than the extracellular metabolites; the correlation between the differential metabolites of *E. coli* was stronger than that of *B. subtilis*, which also indicates that *E. coli* exhibits a violent and tight biochemical reaction to obtain better competitive advantages in the co-culture system.

#### Metabolic pathway analysis

**Fig. 6:**
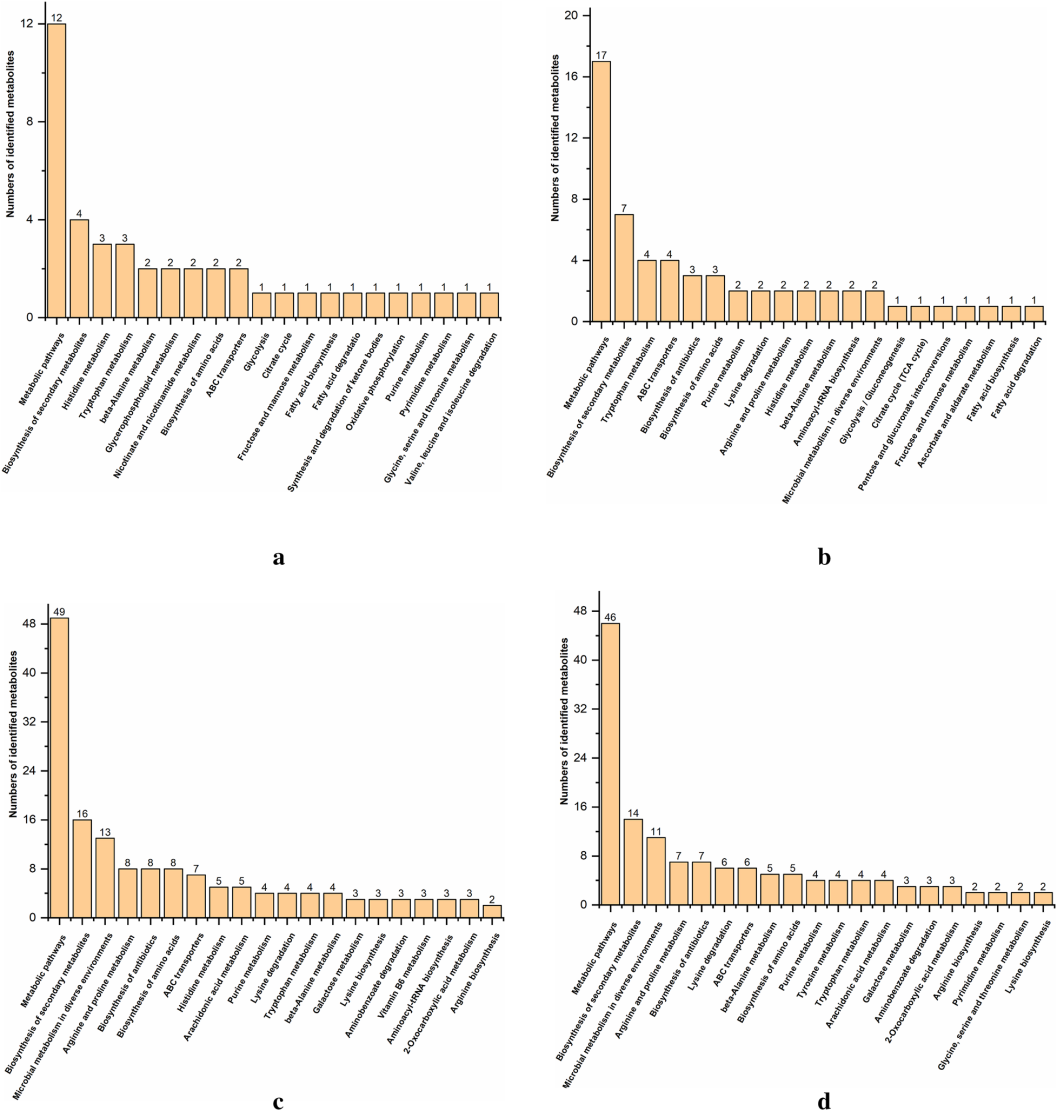
Top 20 KEGG pathway for differential metabolites for group 1 vs 5 (**a**), 2 vs 6 (**b**), 3 vs 5 (**c**), and 4 vs 6 (**d**)

Using KEGG database annotation to identify pathways involved in differential metabolites, based on the mapping results, 44 pathways for extracellular metabolites and 50 pathways for intracellular metabolites of *B. subtilis* were identified, and 72 pathways for extracellular metabolites and 65 pathways for intracellular metabolites of *E. coli* were identified. In addition, the top 20 KEGG pathways for differential metabolite are shown in Fig. 6.

**Fig. 7:**
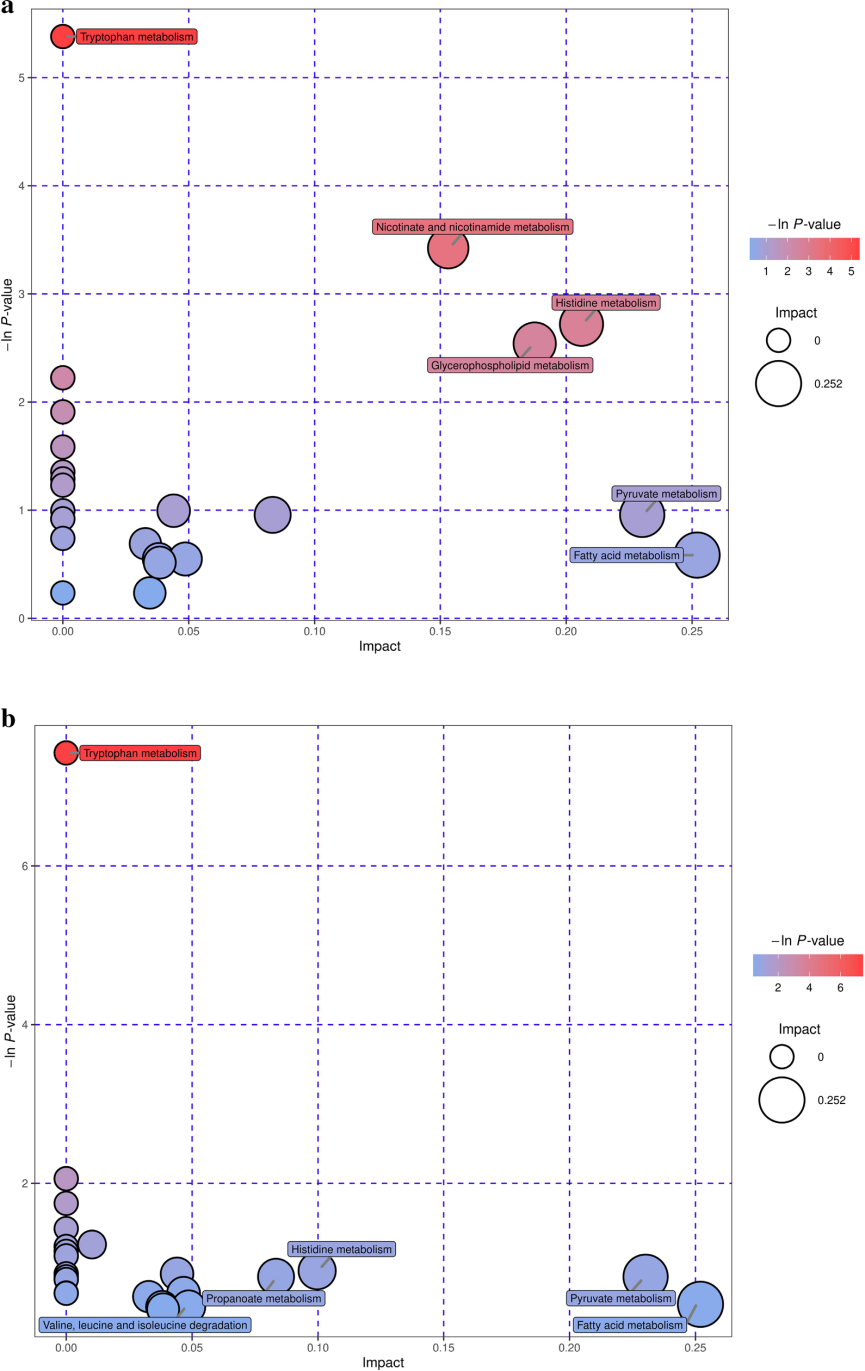

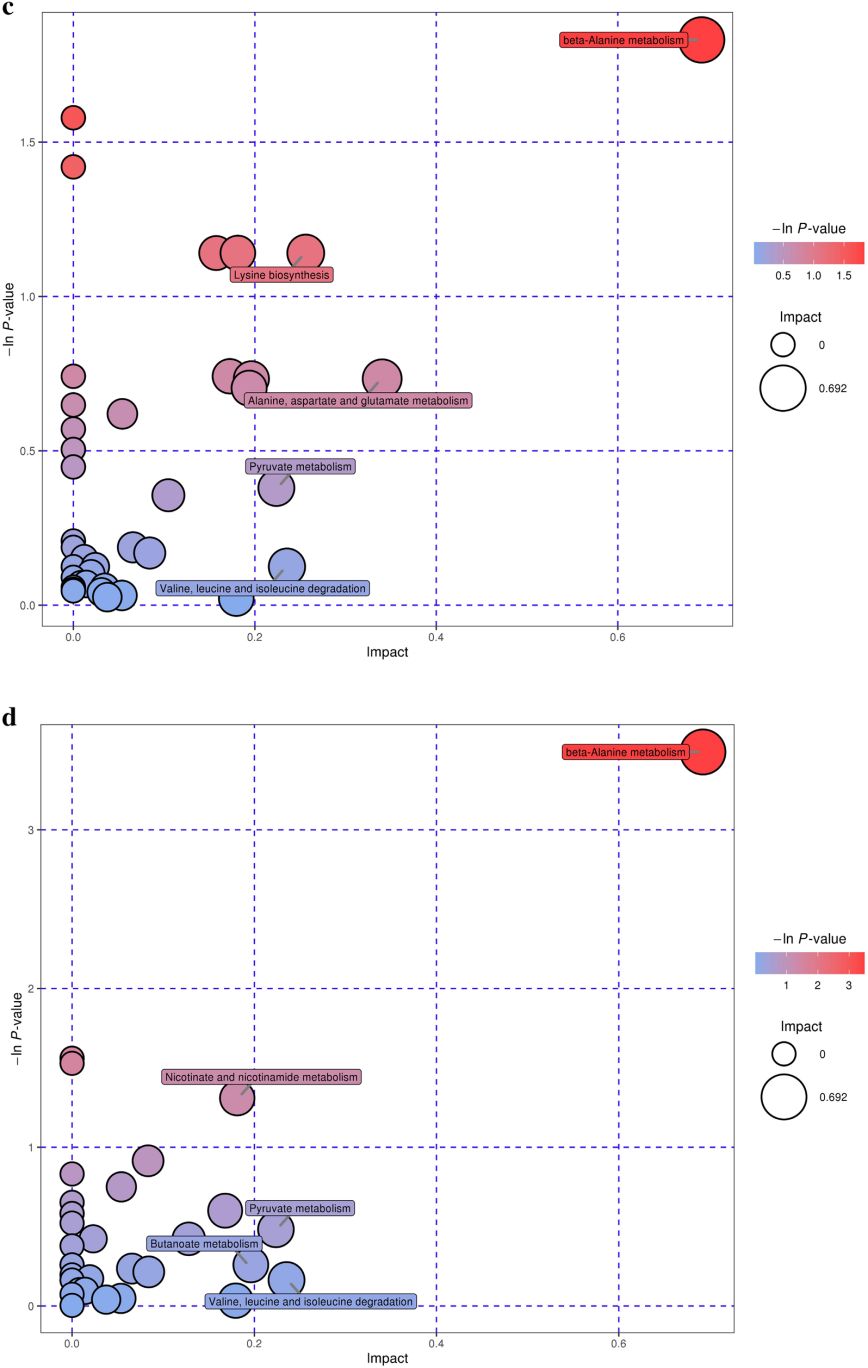
Pathways analysis for recombinant *B. subtilis* and *E. coli*. **a**, **b** extracellular and intracellular pathways of differential metabolites of *B. subtilis*; **c**, **d** extracellular and intracellular pathways of differential metabolites of *E. coli*

Further analysis of the metabolic pathways for differential metabolites could identify the key pathways with the highest correlation with metabolite differences (Additional file 1: Table S3), specifically, through a comprehensive analysis of the pathways in which the differential metabolites are located, including enrichment analysis and topological analysis. The results of the screened pathways are presented in bubble plots. Figure 7a, b demonstrates the selected extracellular and intracellular pathways of *B. subtilis*, while the selected extracellular and intracellular pathways of *E. coli* are shown in Fig. 7c, d. Each bubble in the diagram represents a metabolic pathway. The abscissa and size of the bubble indicate the size of the impact factor of the pathway in the topology analysis, and the ordinate and color of the bubble express the *p* value (taking the negative natural logarithm, namely -ln((*p*)) of the enrichment analysis. It can be seen from the bubble chart that tryptophan metabolism had the highest correlation during the co-fermentation process for *B. subtilis*. Similarly, beta-alanine metabolism had the highest correlation with *E. coli*. It is well known that, tryptophan metabolism is related to protein synthesis. It can participate as a signal molecule in the regulation of synthesis rate (Yanofsky 2007) and is also closely related to carbohydrates, vitamins, and trace elements in the metabolic process (Veldmann et al. 2019). *β*-Alanine metabolism is mainly used to decompose pyrimidine and is related to the formation of metabolic by-products. This also revealed an increase in D-psicose production and the by-product concentrations of formic, acetic, and glycolic acids during the co-culture process in the metabolic pathways. The increase in D-psicose production was due to the improved expression of DTEase during the co-fermentation process, which isomerized more fructose to synthesize D-psicose. As *E. coli* and *B. subtilis* are prone to produce metabolites such as acetic and formic acids during fermentation, the biochemical process is accelerated and closely related to the glycolysis, the pentose phosphate pathway, and the tricarboxylic acid cycle, thereby enhancing the concentration of by-products.

#### Metabolic network analysis

**Fig. 8:**
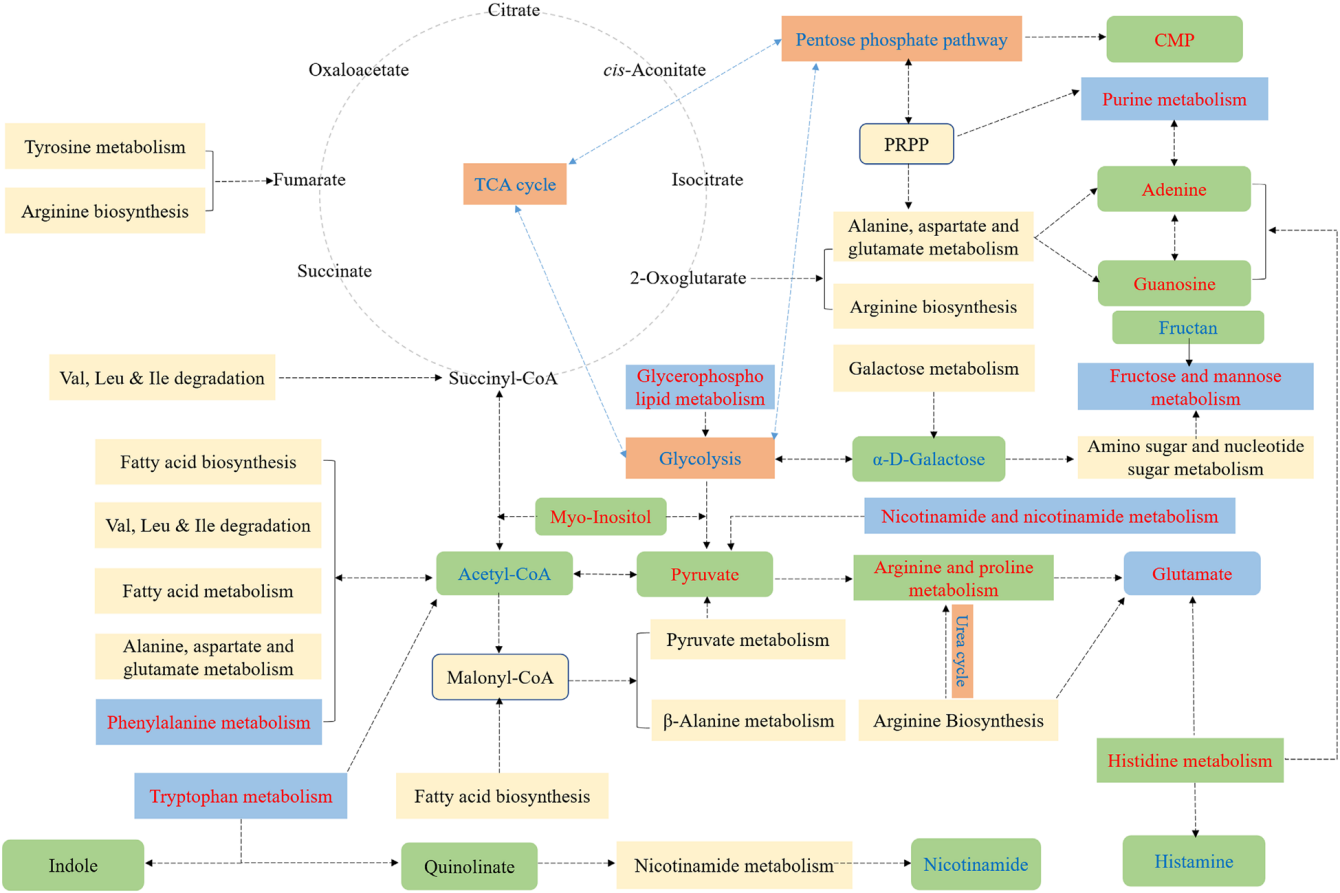
Integration map of some metabolic pathways of recombinant *B. subtilis* and *E. coli* and related differential metabolites. The pathways were obtained from the KEGG database and modified appropriately. Red indicates that some key differential metabolites and metabolic pathways in the process of co-fermentation are upregulated, and similarly blue indicates down-regulation

Metabolic networks describe metabolism and physiological processes in the cell including metabolic reactions and regulatory mechanisms (De Martino et al. 2014), and the results of the network analysis are shown in Additional file 1: Fig. S10. Some integrated metabolic pathways and differential metabolites of the two recombinant strains are shown in Fig. 8. Compared with the mono-cultivation group (Additional file 1: Table S4), the extracellular fructose and mannose metabolism, histidine metabolism, tryptophan metabolism, glycerophospholipid metabolism, and nicotinate and nicotinamide metabolism of *B. subtilis* in the co-culture system showed more prominent changes, and the relationship between them was relatively close. The intracellular purine metabolism, arginine and proline metabolism, histidine metabolism, phenylalanine metabolism, and tryptophan metabolism of *B. subtilis* during co-cultivation showed a strong metabolic reaction to form different regulation nodes.

The metabolic regulation of the co-culture system of *B. subtilis* and *E. coli* was relatively complicated, and the stability and effectiveness of its overall biochemical process were closely related to enzymes, pathways, reactions, and compounds (Weiner et al. 2017). However, compared with the mono-culture group, the regulation of extracellular and intracellular metabolism in the co-culture of *E. coli* was relatively simple, and arginine and proline metabolism showed a clearly changing trend due to the downregulation of key intermediate metabolites. Therefore, in the co-cultivation process of recombinant strains, it was obvious that the metabolic reactions of *B. subtilis* were drastically altered, including multiple pathways, while the biochemical process of *E. coli* was relatively simple. Furthermore, in the co-culture system, *B. subtilis* and *E. coli* needed to enhance stress tolerance to balance the cells to reduce damage in response to environmental stress, and changing the lipid composition of cell components to regulate membrane fluidity may be a defence mechanism (Valluru and Van den Ende 2011). For example, myo-inositol is a precursor substance of many compounds, a type of inositol derivative formed after lipid-dependent phosphorylation, which is both an important membrane structure and signal substance (Li et al. 2021). In this study, the upregulation of myo-inositol abundance in the co-culture system provides more precursors for lipid synthesis to achieve a cellular stress response. These beneficial results provide a metabolic regulation basis for subsequent co-culture systems.

## Conclusions

Co-production of D-psicose and lipase by co-cultivation of engineered *B. subtilis* and *E. coli* was achieved for the first time together with 11.70 g/L of D-psicose and 16.03 U/mg of recombinant lipase. The effective utilization of glucose and fructose was also realized through metabolomics analysis, and 684 differential metabolites were identified; the relative content of co-culture metabolites was significantly higher than that of mono-culture metabolites. In addition, tryptophan metabolism and *β*-alanine metabolism were found to have the highest correlation during the co-culture bioprocess. Metabolic regulation of the co-culture system is relatively complicated, and the efficiency and stability of mixed bacteria is the overall biochemical process closely related to enzymes, pathways, reactions, and compounds.

### Supplementary Information

**Additional file 1. Table S1.** Groups information of metabolomics samples. **Table S2.** PCA and OPLS-DA model parameters. **Table S3.** Comparisons of differential metabolites between engineered *B. subtilis* and *E. coli* in certain important metabolic pathways. **Table S4. **Metabolic network analysis of engineered *B. subtilis* and *E. coli*. **Fig. S1.** HSQC analysis of the standard D-psicose (**a**), D-fructose (**b**), and D-glucose (**c**). **Fig. S2. **Score scatter plot for PCA model with QC. **Fig. S3.** Score scatter plot of PCA (**a**), OPLS-DA model (**b**), permutation test (**c**), and volcano plot (**d**) for group **1 **vs** 5. Fig. S4.** Score scatter plot of PCA (**a**), OPLS-DA model (**b**), permutation test (**c**), and volcano plot (**d**) for group **3 **vs** 5. Fig. S5.** Score scatter plot of PCA (**a**), OPLS-DA model (**b**), permutation test (**c**), and volcano plot (**d**) for group **2 **vs** 6. Fig. S6.** Score scatter plot of PCA (**a**), OPLS-DA model (**b**), permutation test (**c**), and volcano plot (**d**) for group **4 **vs** 6**. **Fig. S7.** Heatmap of hierarchical cluster analysis for extracellular and intracellular differential metabolites. **a** extracellular differential metabolites between* B. subtilis* and mixed strains in; **b** intracellular differential metabolites between *B. subtilis* and mixed strains; **c** extracellular differential metabolites of *E. coli* and mixed strains;** d** intracellular differential metabolites of *E. coli* and mixed strains.** Fig. S8.** Radar char analysis for differential metabolites of group 1 vs 5 (**a**), 2 vs 6 (**b**), 3 vs 5 (**c**), and 4 vs 6 (**d**). **Fig. S9.** Correlation analysis for differential metabolites of group 1 vs 5 (**a**), 2 vs 6 (**b**), 3 vs 5 (**c**), and 4 vs 6 (**d**). **Fig. S10.** Network analysis for group 1 vs 5 (**a**), 2 vs 6 (**b**), 3 vs 5 (**c**), and 4 vs 6 (**d**).

## Data Availability

The raw/processed data required to reproduce these findings cannot be shared at this time as the data also forms part of an ongoing study.
