# Supplementary material for: A novel strategy for D-psicose and lipase co-production using a co-culture system of engineered Bacillus subtilis and Escherichia coli and bioprocess analysis using metabolomics
Source: Bioresour Bioprocess. 2021 Aug 19;8(1):77. doi: 10.1186/s40643-021-00429-8 (PMC10992840; doi:10.1186/s40643-021-00429-8)
Supplement: Supplementary file 1 — Additional file 1. Table S1. Groups information of metabolomics samples. Table S2. PCA and OPLS-DA model parameters. Table S3. Comparisons of differential metabolites between engineered B. subtilis and E. coli in certain important metabolic pathways. Table S4. Metabolic network analysis of engineered B. subtilis and E. coli. Fig. S1. HSQC analysis of the standard D-psicose (a), D-fructose (b), and D-glucose (c). Fig. S2. Score scatter plot for PCA model with QC. Fig. S3. Score scatter plot of PCA (a), OPLS-DA model (b), permutation test (c), and volcano plot (d) for group 1 vs 5. Fig. S4. Score scatter plot of PCA (a), OPLS-DA model (b), permutation test (c), and volcano plot (d) for group 3 vs 5. Fig. S5. Score scatter plot of PCA (a), OPLS-DA model (b), permutation test (c), and volcano plot (d) for group 2 vs 6. Fig. S6. Score scatter plot of PCA (a), OPLS-DA model (b), permutation test (c), and volcano plot (d) for group 4 vs 6. Fig. S7. Heatmap of hierarchical cluster analysis for extracellular and intracellular differential metabolites. a extracellular differential metabolites between B. subtilis and mixed strains in; b intracellular differential metabolites between B. subtilis and mixed strains; c extracellular differential metabolites of E. coli and mixed strains; d intracellular differential metabolites of E. coli and mixed strains. Fig. S8. Radar char analysis for differential metabolites of group 1 vs 5 (a), 2 vs 6 (b), 3 vs 5 (c), and 4 vs 6 (d). Fig. S9. Correlation analysis for differential metabolites of group 1 vs 5 (a), 2 vs 6 (b), 3 vs 5 (c), and 4 vs 6 (d). Fig. S10. Network analysis for group 1 vs 5 (a), 2 vs 6 (b), 3 vs 5 (c), and 4 vs 6 (d). [file 40643_2021_429_MOESM1_ESM.docx]

**Supplementary Materials**

**A novel strategy for D-psicose and lipase co-production using a co-culture system of engineered *Bacillus subtilis* and *Escherichia coli* and bioprocess analysis using metabolomics**

Jun Zhang ^a, b, d^, Wen Luo ^a^, Zhiyuan Wang ^a^, Xiaoyan Chen ^a, d^, Pengmei Lv ^a ^[[1]](#footnote-1)^*^, Jingliang Xu ^a, c *^

*^a^* *Guangzhou Institute of Energy Conversion, Chinese Academy of Sciences, CAS Key Laboratory of Renewable Energy, Guangdong Provincial Key Laboratory of New and Renewable Energy Research and Development**, Guangzhou 510640, China*

*^b^ College of Food Science and Technology, Guangdong Ocean University, Guangdong Provincial Key Laboratory of Aquatic Product Processing and Safety, Guangdong Province Engineering Laboratory for Marine Biological Products, Guangdong Provincial Engineering Technology Research Center of Seafood, Key Laboratory of Advanced Processing of Aquatic Product of Guangdong Higher Education Institution, Zhanjiang 524088, China
^c^ School of Chemical Engineering, Zhengzhou University, Zhengzhou, 450001, China*

*^d^ University of Chinese Academy of Sciences, Beijing 100049, China*

**Tables**

**Table S1** Groups information of metabolomics samples

| Groups | Serial numbers description | Groups | Serial numbers description |
| --- | --- | --- | --- |
| 1 | Extracellular metabolites of *B. subtilis* 24 h-1 | 4 | Intracellular metabolites of *E. coli* 24 h-1 |
| 1 | Extracellular metabolites of *B. subtilis* 24 h-2 | 4 | Intracellular metabolites of *E. coli* 24 h-2 |
| 1 | Extracellular metabolites of *B. subtilis* 24 h-3 | 4 | Intracellular metabolites of *E. coli* 24 h-3 |
| 1 | Extracellular metabolites of *B. subtilis* 24 h-4 | 4 | Intracellular metabolites of *E. coli* 24 h-4 |
| 2 | Intracellular metabolites of *B. subtilis* 24 h-1 | 5 | Extracellular metabolites of *B. subtilis* and *E. coli* 24 h-1 |
| 2 | Intracellular metabolites of *B. subtilis* 24 h-2 | 5 | Extracellular metabolites of *B. subtilis* and *E. coli* 24 h-2 |
| 2 | Intracellular metabolites of *B. subtilis* 24 h-3 | 5 | Extracellular metabolites of *B. subtilis* and *E. coli* 24 h-3 |
| 2 | Intracellular metabolites of *B. subtilis* 24 h-4 | 5 | Extracellular metabolites of *B. subtilis* and *E. coli* 24 h-4 |
| 3 | Extracellular metabolites of *E. coli* 24 h-1 | 6 | Intracellular metabolites of *B. subtilis* and *E. coli* 24h-1 |
| 3 | Extracellular metabolites of *E. coli* 24 h-2 | 6 | Intracellular metabolites of *B. subtilis* and *E. coli* 24h-2 |
| 3 | Extracellular metabolites of *E. coli* 24 h-3 | 6 | Intracellular metabolites of *B. subtilis* and *E. coli* 24h-3 |
| 3 | Extracellular metabolites of *E. coli* 24 h-4 | 6 | Intracellular metabolites of *B. subtilis* and *E. coli* 24h-4 |

**Table S2** PCA and OPLS-DA model parameters

| Model | Type | A ^a^ | N ^b^ | R^2^X(cum) ^c^ | R^2^Y(cum) ^d^ | Q^2^(cum) ^e^ | Title ^f^ |
| --- | --- | --- | --- | --- | --- | --- | --- |
| 1 | PCA | 3 | 28 | 0.666 |  |  | TOTAL with QC |
| 2 | PCA | 3 | 24 | 0.709 |  |  | TOTAL |
| 3 | PCA | 3 | 8 | 0.794 |  |  | 1-5 |
| 4 | PCA | 3 | 8 | 0.872 |  |  | 3-5 |
| 5 | PCA | 3 | 8 | 0.803 |  |  | 2-6 |
| 6 | PCA | 3 | 8 | 0.882 |  |  | 4-6 |
| 7 | OPLS-DA | 1+1+0 | 8 | 0.558 | 0.996 | 0.701 | 1-5 |
| 8 | OPLS-DA | 1+1+0 | 8 | 0.621 | 0.992 | 0.841 | 3-5 |
| 9 | OPLS-DA | 1+1+0 | 8 | 0.541 | 0.997 | 0.701 | 2-6 |
| 10 | OPLS-DA | 1+1+0 | 8 | 0.658 | 0.987 | 0.928 | 4-6 |

^a^: the number of principal components of the model; ^b^: the number of observations of the model (here is the number of samples); ^c^: represents the explanatory power of the model to X variables; ^d^: represents the explanatory power of the model to the Y variable; ^e:^ the predictability of the model; ^f^: the data object corresponding to the model.

**Table S3** Comparisons of differential metabolites between engineered *B. subtilis* and *E. coli* in certain important metabolic pathways

| Metabolic pathways | Extracellular metabolites | Intracellular metabolites |
| --- | --- | --- |
| Tryptophan metabolism | Indoleacetaldehyde, 5-Hydroxyindoleacetaldehyde, Acetyl-CoA | 3-Hydroxyanthranilic acid, Indoleacetaldehyde, 5-Hydroxyindoleacetaldehyde, Acetyl-CoA |
| Nicotinate and nicotinamide metabolism | NAD, Niacinamide | NAD, Niacinamide |
| Histidine metabolism | L-Histidine, L-Histidinol, 4-Imidazolone-5-propionic acid, Urocanic acid, L-Glutamic acid, Imidazoleacetic acid | L-Histidine, 4-Imidazolone-5-propionic acid, Urocanic acid |
| beta-Alanine metabolism | Beta-Alanine, 3-Aminopropionaldehyde | Beta-Alanine, 3-Aminopropionaldehyde, Pantothenic acid |
| Nitrogen metabolism | L-Asparagine, L-Glutamic acid, Adenosine monophosphate | L-Histidine, Adenosine monophosphate |
| Glycerophospholipid metabolism | Glycerophosphocholine, Choline |  |
| Arginine and proline metabolism | N-Acetyl-L-glutamate 5-semialdehyde, L-Proline | Ornithine, N-Acetyl-L-glutamate 5-semialdehyde, L-Proline, 4-Aminobutyraldehyde |
| Purine metabolism | Adenine, Adenosine monophosphate, Adenosine, Cyclic AMP, Cyclic GMP | Adenine, Guanosine, Adenosine monophosphate, Adenosine, Cyclic AMP, Cyclic GMP |
| Galactose metabolism |  | Melibiose |

Note: the differential metabolites present in each column have relatively high contents.

**Table S4** Metabolic network analysis of engineered *B. subtilis* and *E. coli*

| KEGG.id | Entry. type | KEGG.name | *p*. score |
| --- | --- | --- | --- |
| 1 vs 5 |  |  |  |
| bsu00051 | pathway | Fructose and mannose metabolism | 0.040209749 |
| bsu00340 | pathway | Histidine metabolism | 0.000001 |
| bsu00380 | pathway | Tryptophan metabolism | 0.011133822 |
| bsu00564 | pathway | Glycerophospholipid metabolism | 0.000171471 |
| bsu00760 | pathway | Nicotinate and nicotinamide metabolism | 0.048772495 |
| 3 vs 5 |  |  |  |
| bsu00330 | pathway | Arginine and proline metabolism | 0.000001 |
| bsu00472 | pathway | D-Arginine and D-ornithine metabolism | 0.000001 |
| 2 vs 6 |  |  |  |
| eco00340 | pathway | Histidine metabolism | 0.000143089 |
| eco00380 | pathway | Tryptophan metabolism | 0.000237612 |
| 4 vs 6 |  |  |  |
| eco00330 | pathway | Arginine and proline metabolism | 0.000001 |

**Figures**


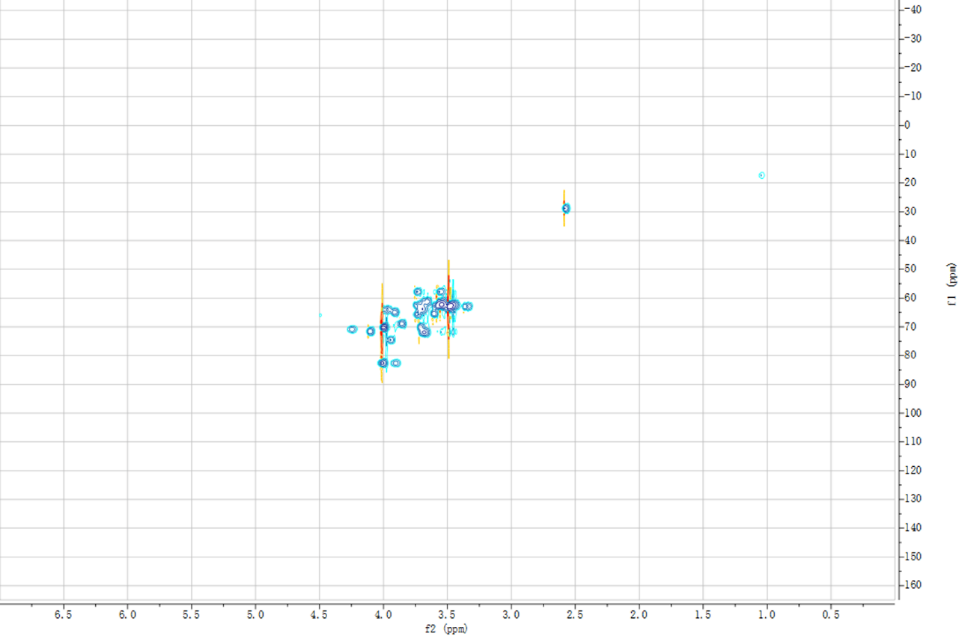


(a)


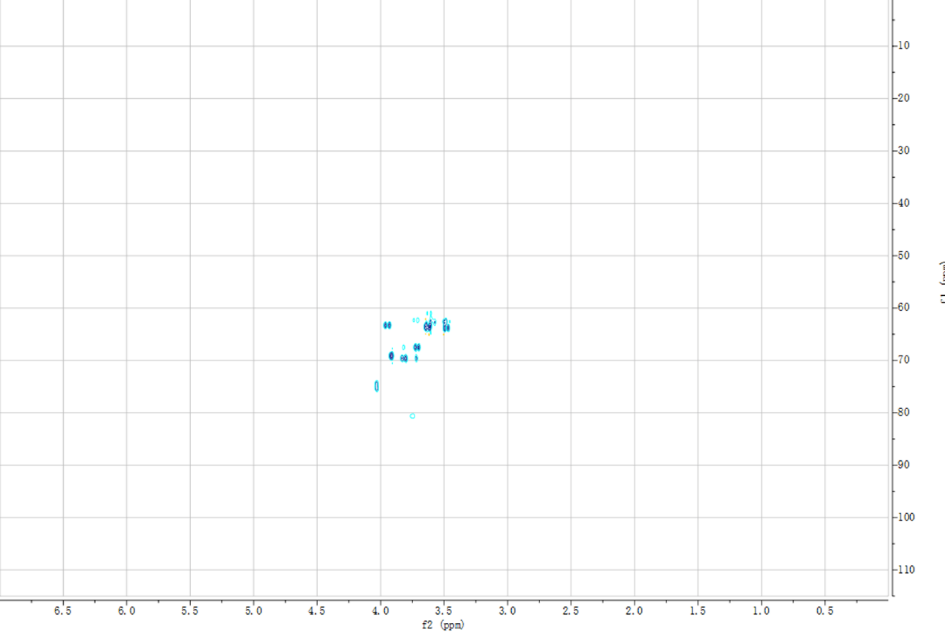


(b)


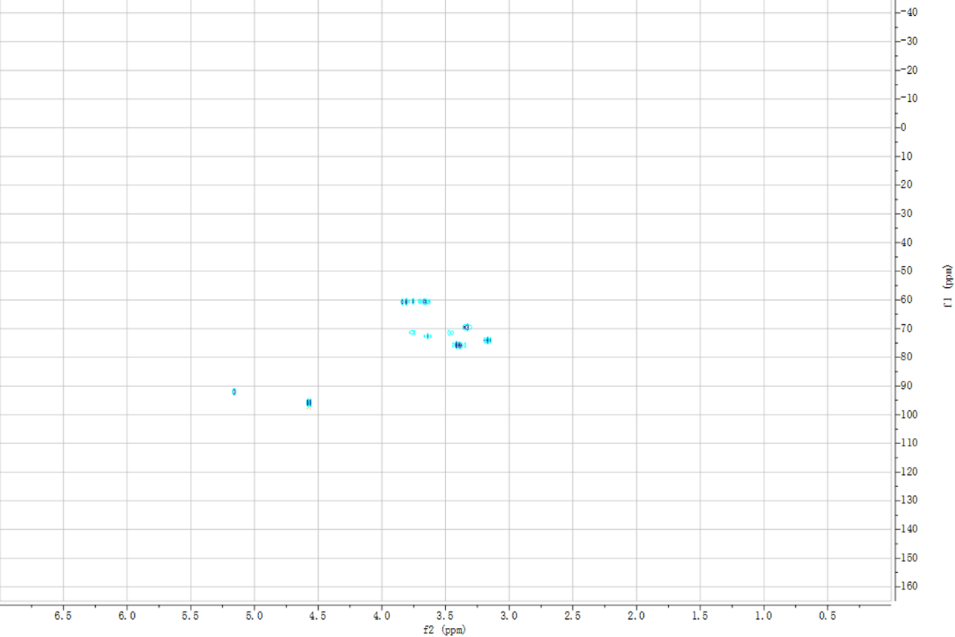


(c)

**Fig. S1** HSQC analysis of the standard D-psicose (a), D-fructose (b), and D-glucose (c).


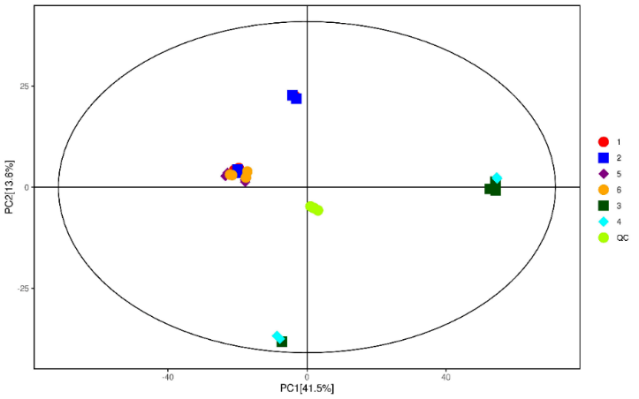


**Fig. S2** Score scatter plot for PCA model with QC.


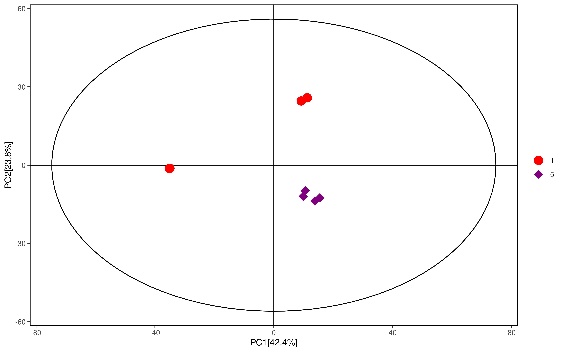

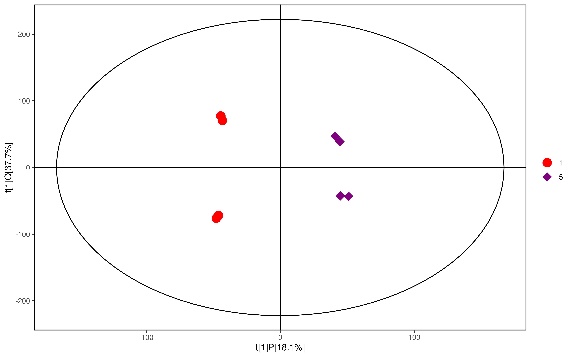


(b)

(a)

(c)


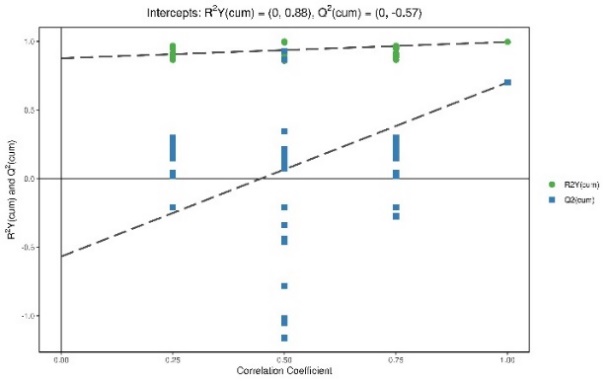

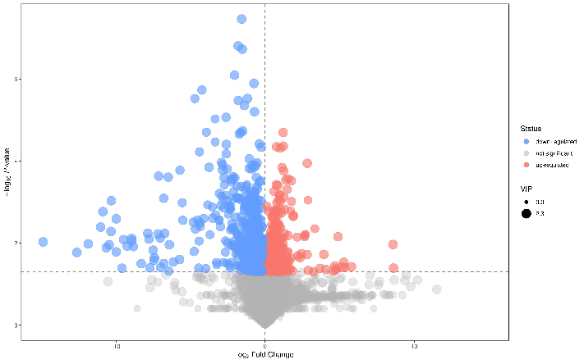


(d)

**Fig. S3** Score scatter plot of PCA (a), OPLS-DA model (b), permutation test (c), and volcano plot (d) for group **1** vs **5.**


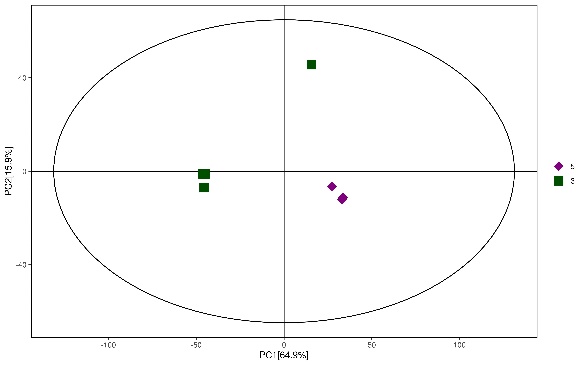

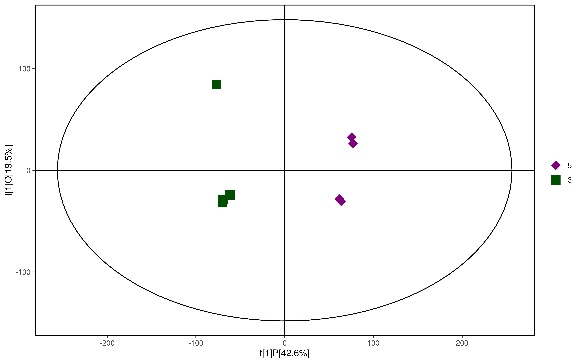


(b)

(a)


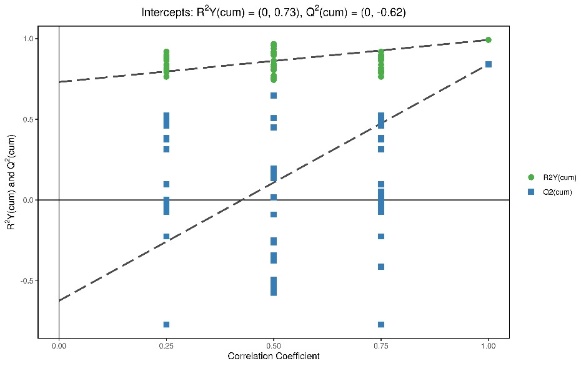

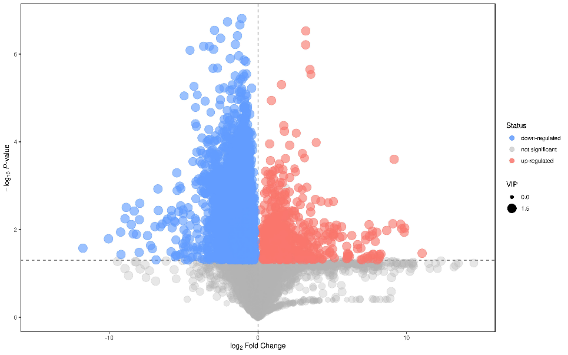


(d)

(c)

**Fig. S4** Score scatter plot of PCA (a), OPLS-DA model (b), permutation test (c), and volcano plot (d) for group **3** vs **5.**


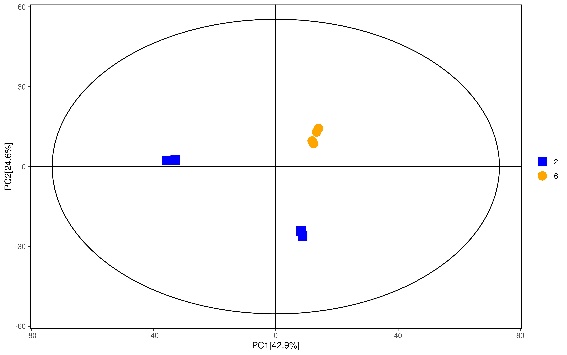

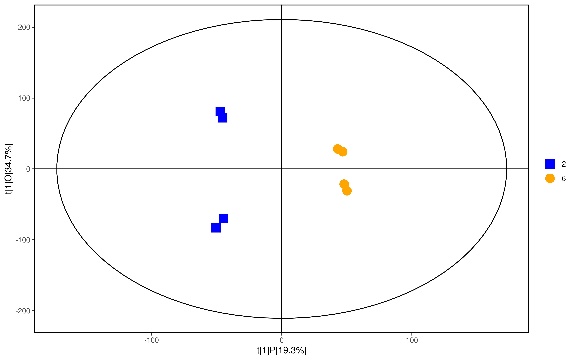


(c)

(d)

(b)

(a)


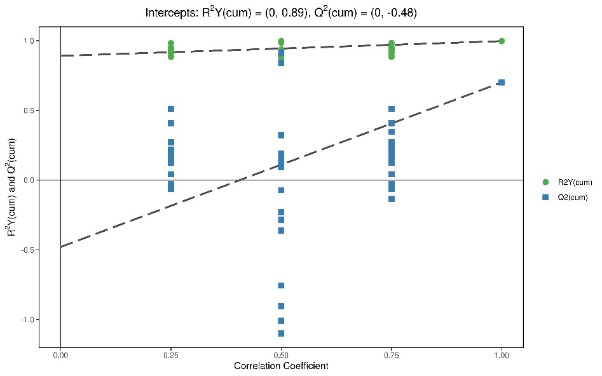

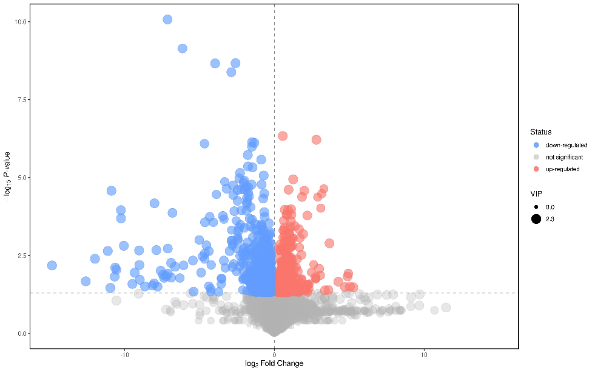


**Fig. S5** Score scatter plot of PCA (a), OPLS-DA model (b), permutation test (c), and volcano plot (d) for group **2** vs **6.**


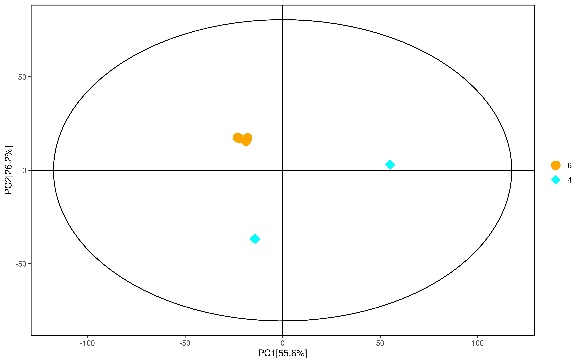

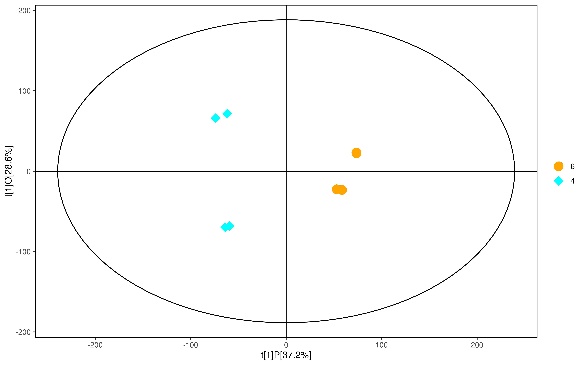


(b)

(a)

(c)


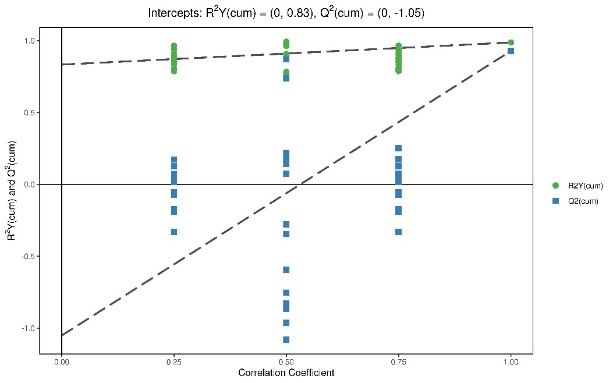

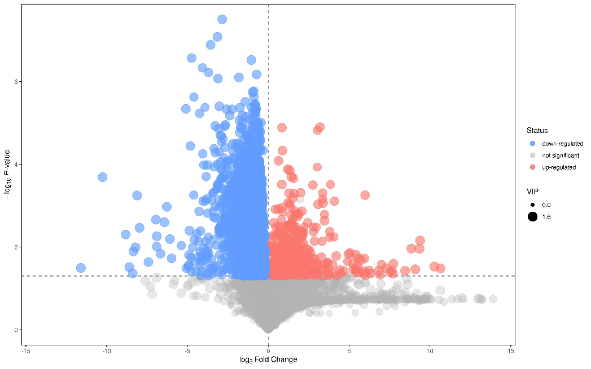


(d)

**Fig. S6** Score scatter plot of PCA (a), OPLS-DA model (b), permutation test (c), and volcano plot (d) for group **4** vs **6**


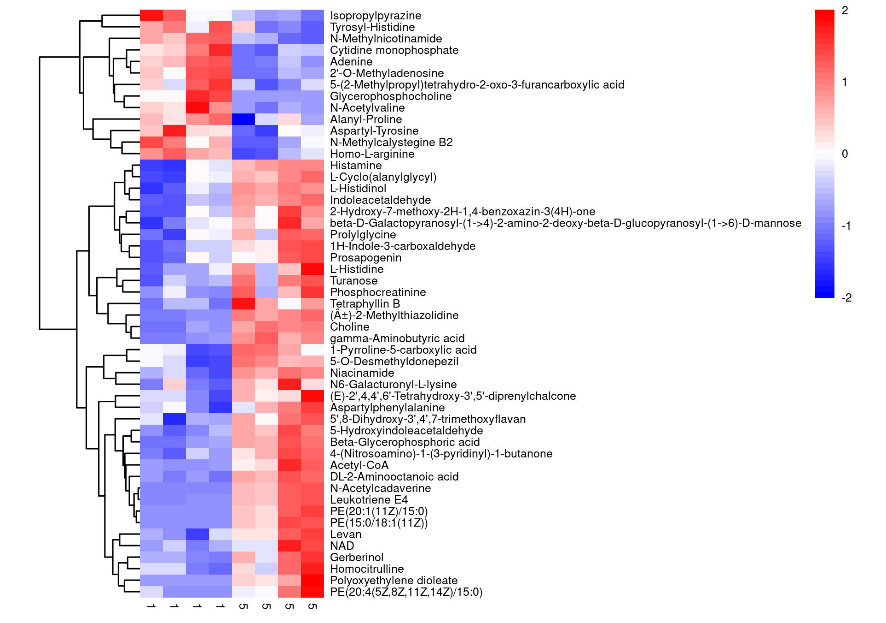


(a)


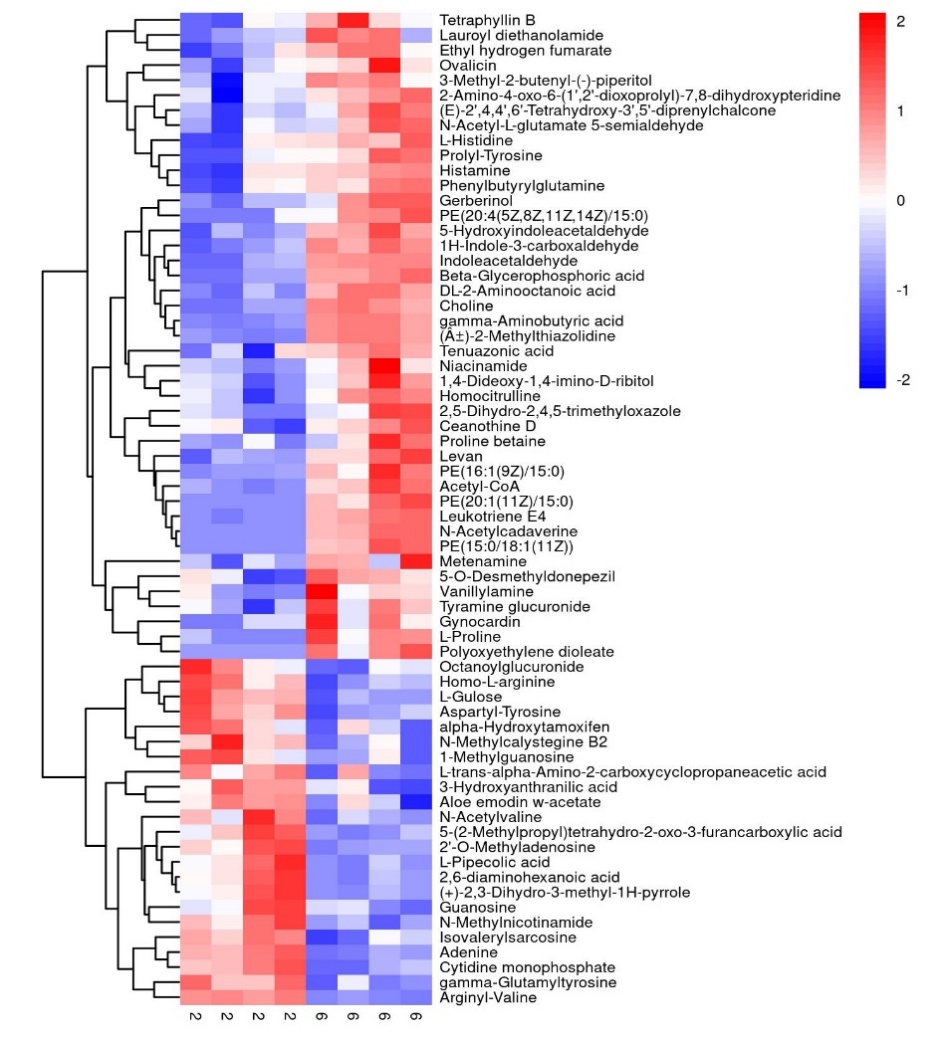


(b)


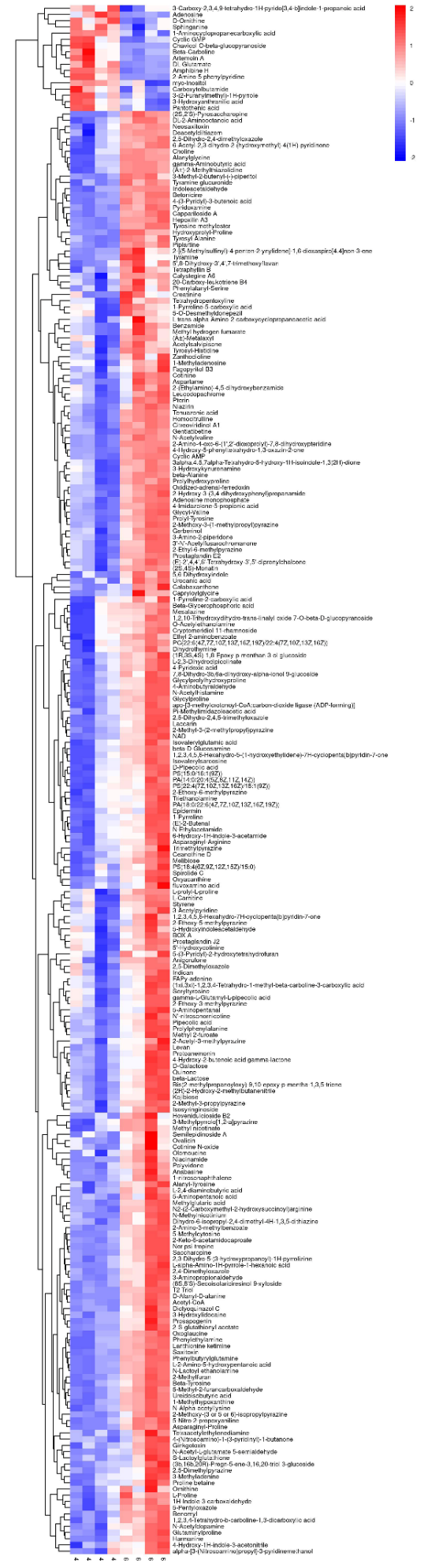


(c)


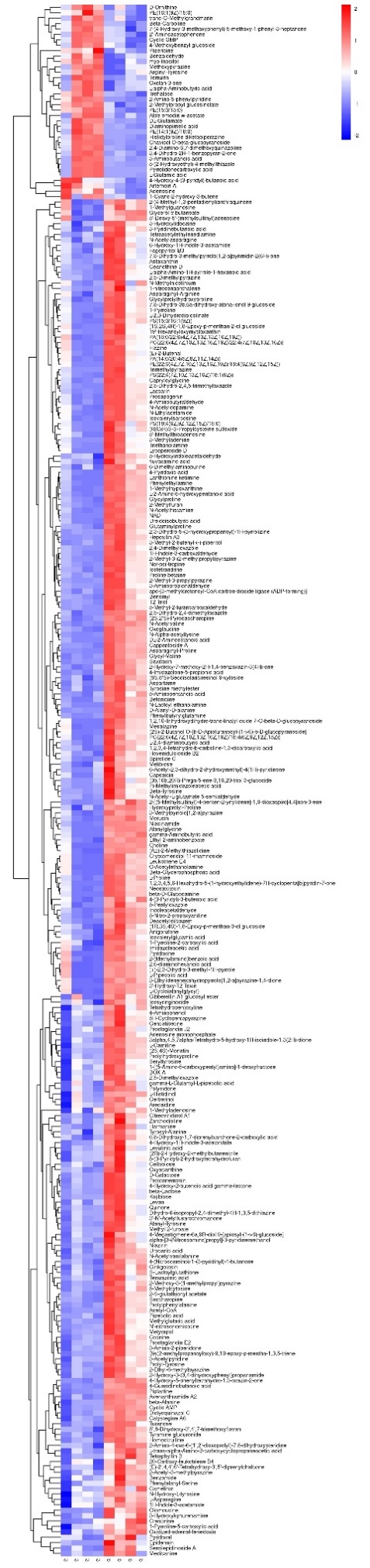
**Fig. S7** Heatmap of hierarchical cluster analysis for extracellular and intracellular differential metabolites. **a**. extracellular differential metabolites between *B. subtilis* and mixed strains in; **b**. intracellular differential metabolites between *B. subtilis* and mixed strains; **c**. extracellular differential metabolites of *E. coli* and mixed strains; **d**. intracellular differential metabolites of *E. coli* and mixed strains.

(d)


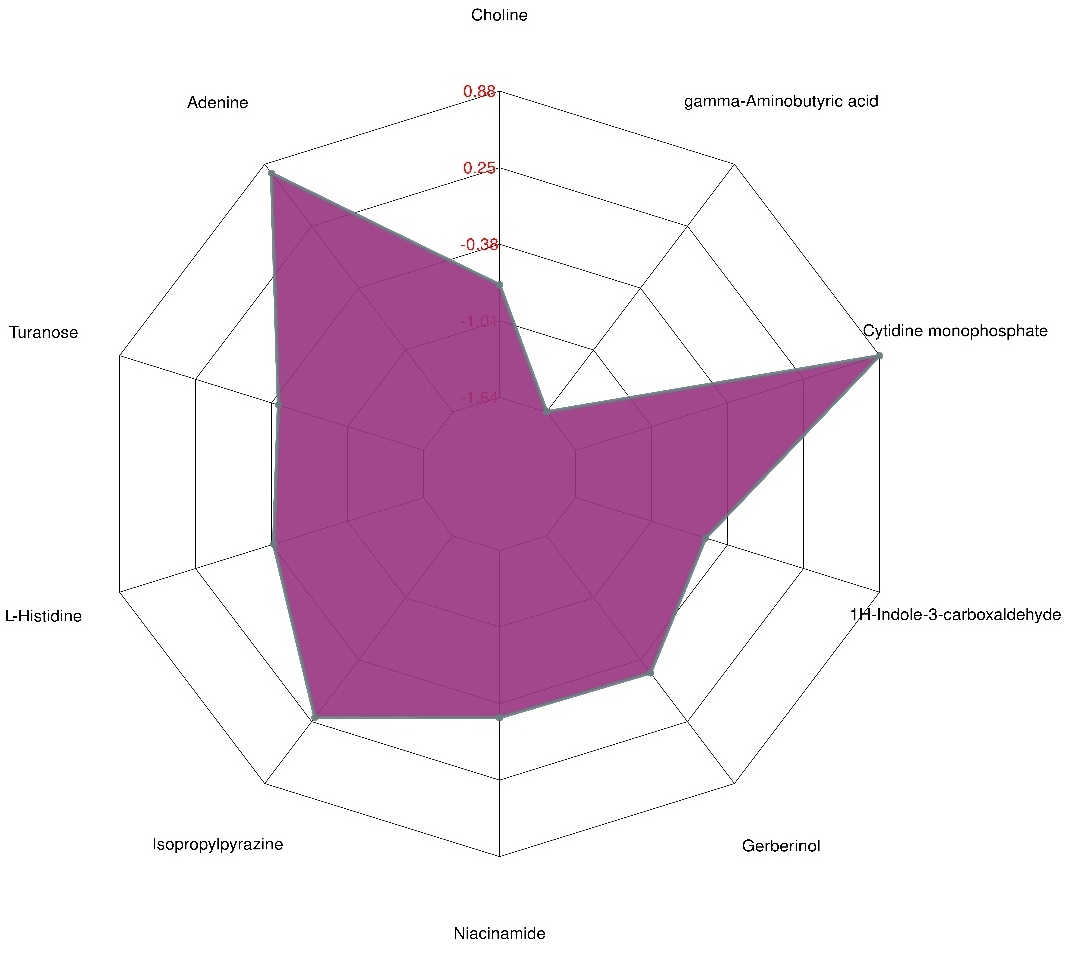

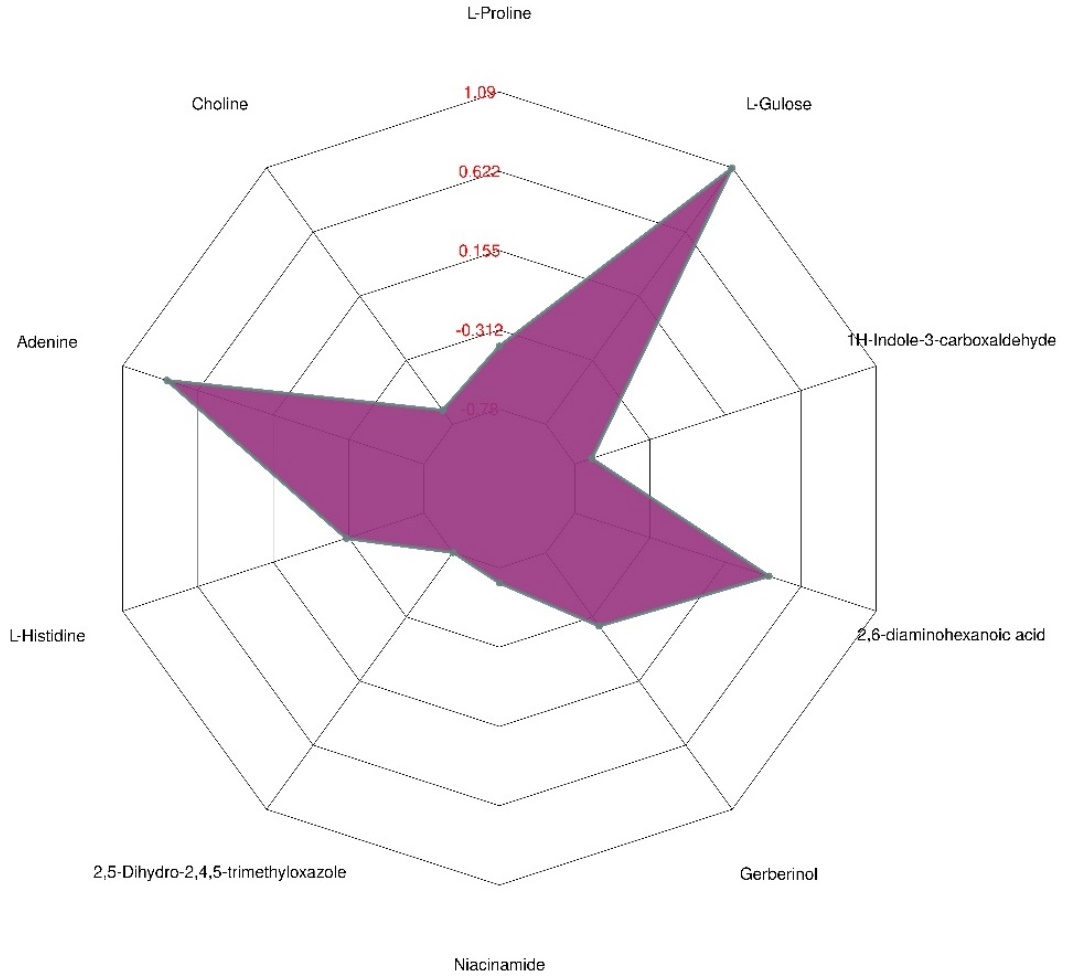


(b)

(a)


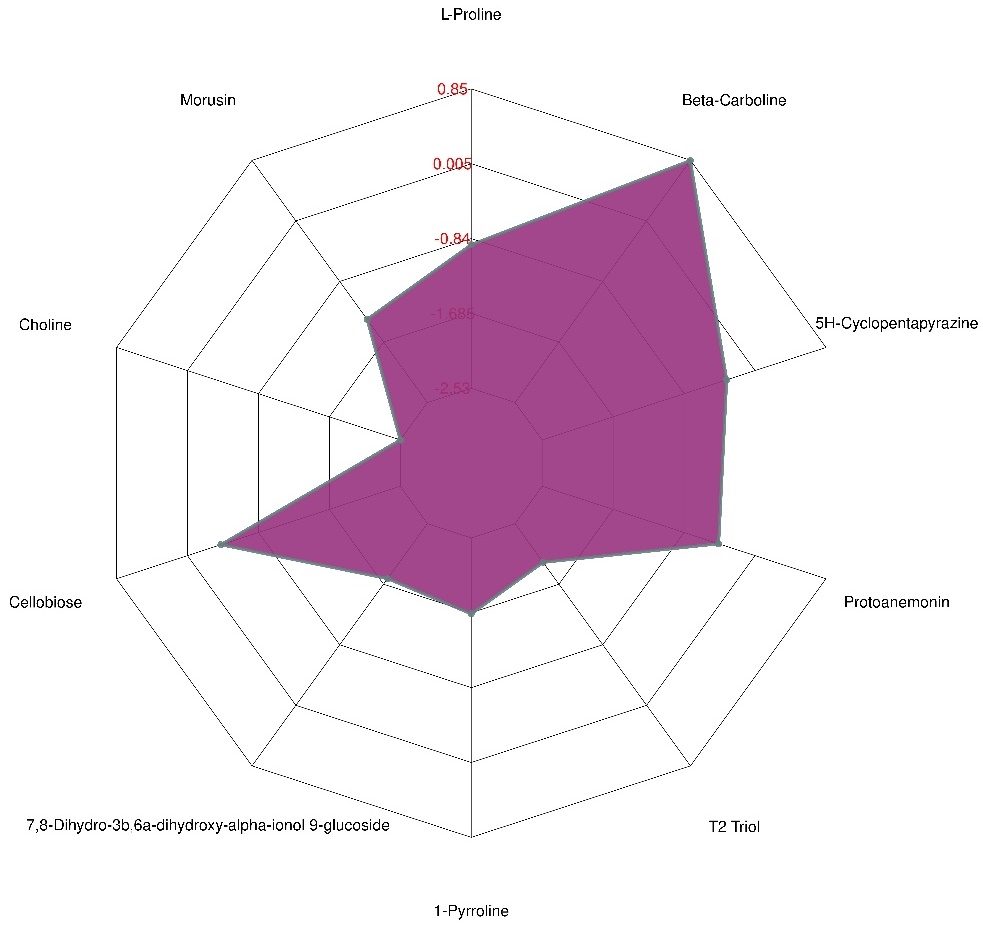

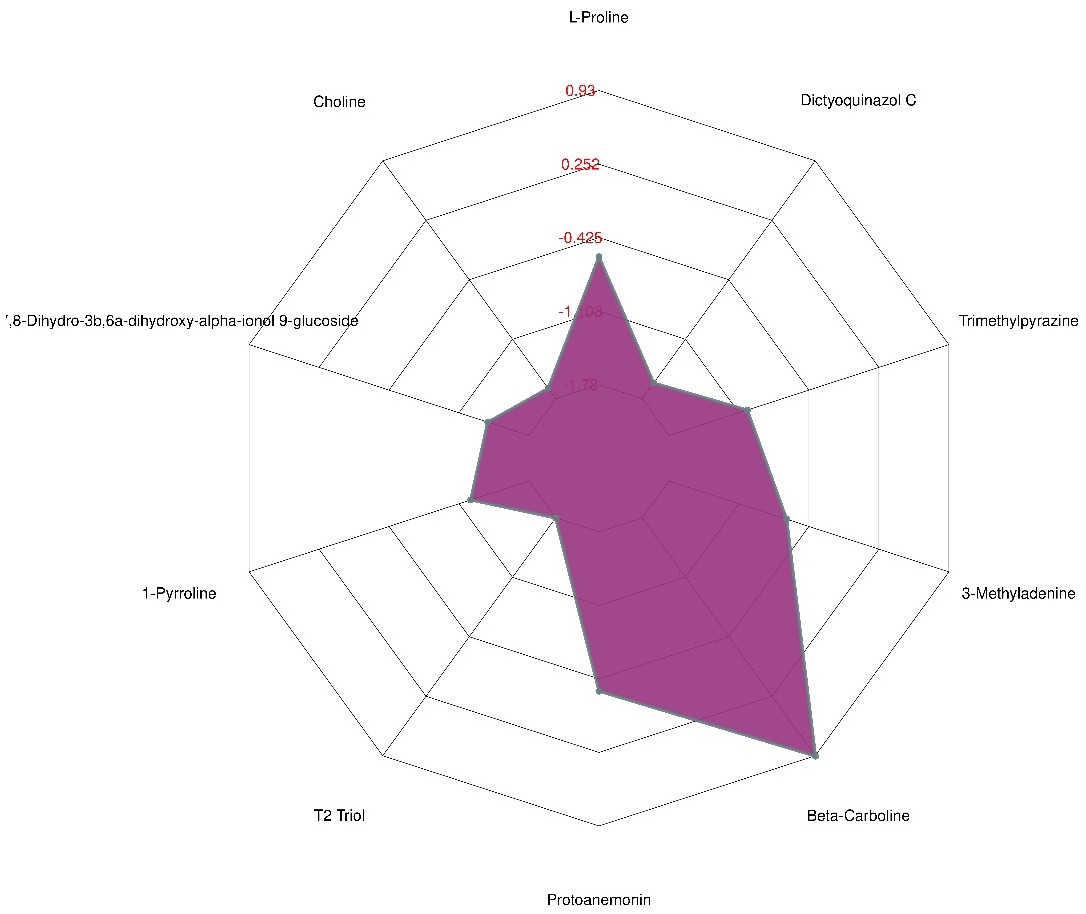


(d)

(c)

**Fig. S8** Radar char analysis for differential metabolites of group 1 vs 5 (a), 2 vs 6 (b), 3 vs 5 (c), and 4 vs 6 (d).


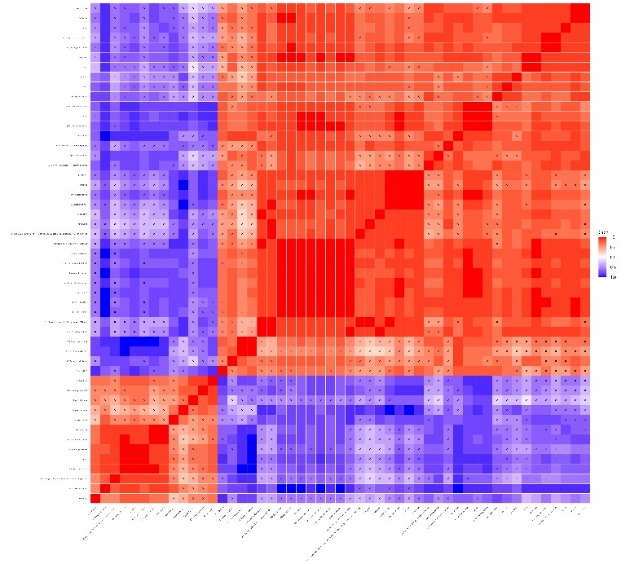

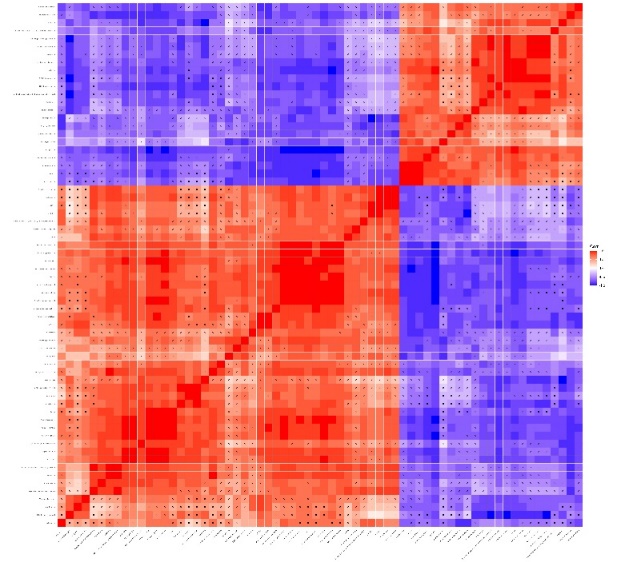


(a)

(b)


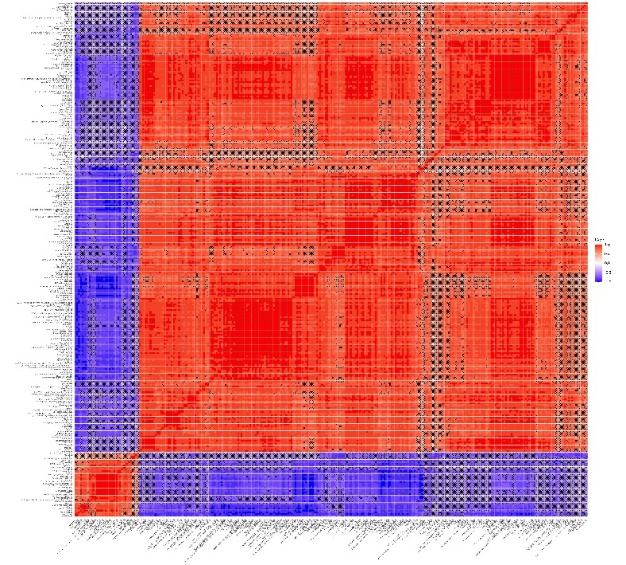

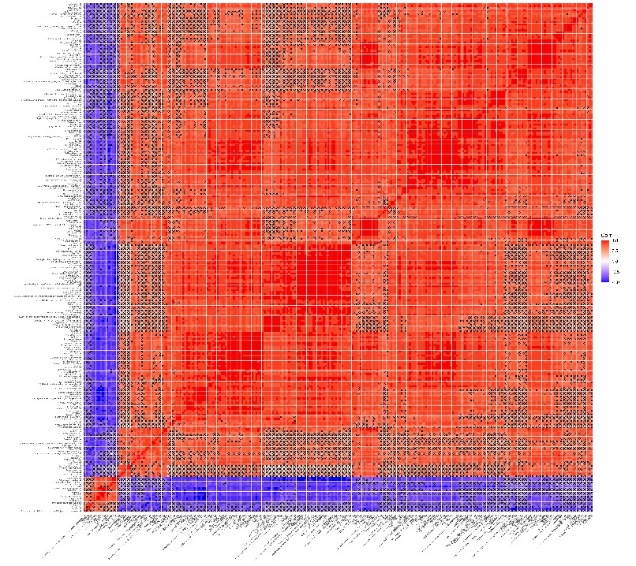


(c)

(d)

**Fig. S9** Correlation analysis for differential metabolites of group 1 vs 5 (a), 2 vs 6 (b), 3 vs 5 (c), and 4 vs 6 (d).


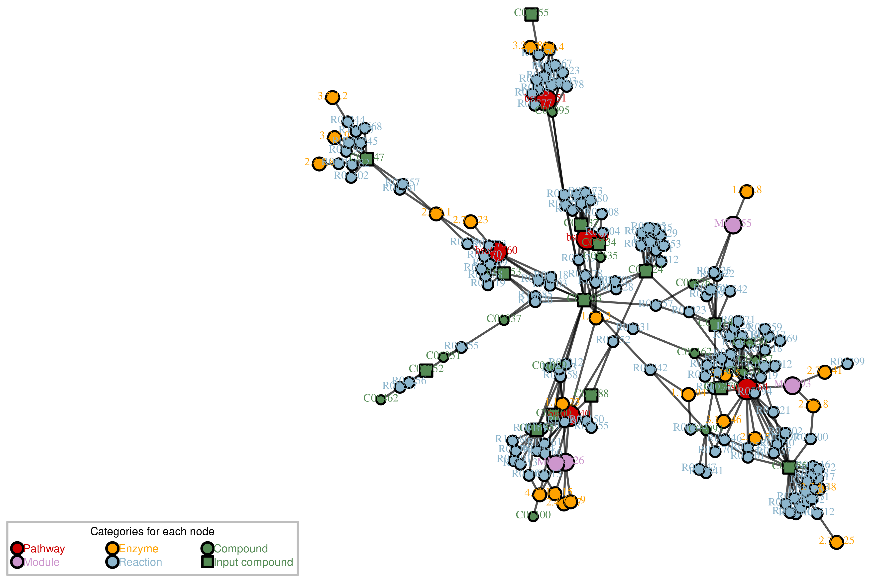

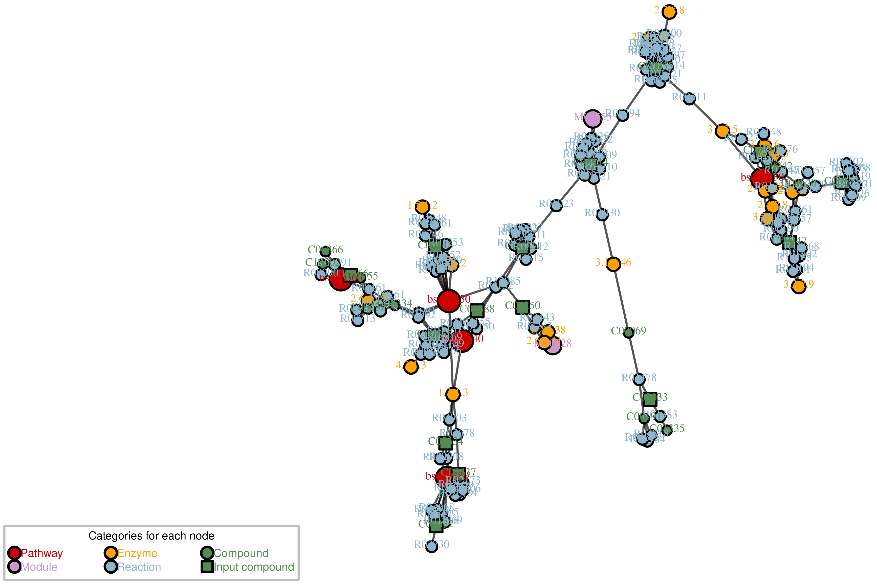


(b)

(a)


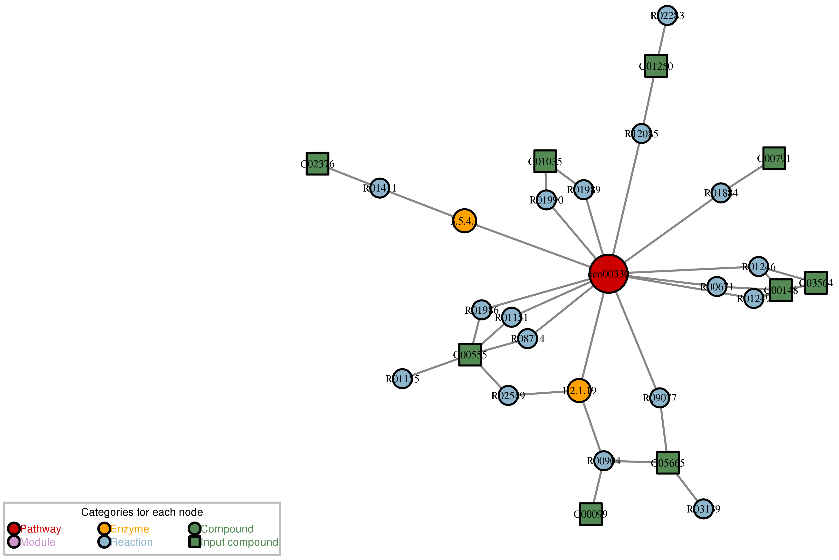

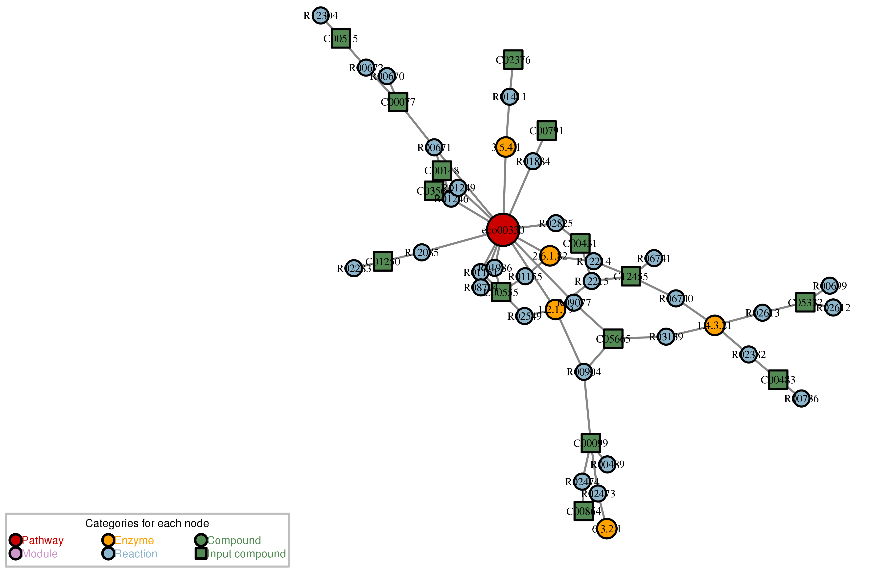


(d)

(c)

**Fig. S10** Network analysis for group 1 vs 5 (a), 2 vs 6 (b), 3 vs 5 (c), and 4 vs 6 (d).

1. *Corresponding Author. Tel./Fax: +86 020 87057760

   E-mail address: [lvpm@ms.giec.ac.cn](mailto:lvpm@ms.giec.ac.cn%20(P); [xujl@zzu.edu.cn](mailto:xujl@zzu.edu.cn). [↑](#footnote-ref-1)
